# Stationary distribution of a reaction-diffusion hepatitis B virus infection model driven by the Ornstein-Uhlenbeck process

**DOI:** 10.1371/journal.pone.0292073

**Published:** 2023-09-29

**Authors:** Zhenyu Zhang, Guizhen Liang, Kangkang Chang

**Affiliations:** 1 Academy of Fine Arts, Xinxiang University, Xinxiang, P.R. China; 2 School of Mathematics and Statistics, Xinxiang University, Xinxiang, P.R. China; United Arab Emirates University, UNITED ARAB EMIRATES

## Abstract

A reaction-diffusion hepatitis B virus (HBV) infection model based on the mean-reverting Ornstein-Uhlenbeck process is studied in this paper. We demonstrate the existence and uniqueness of the positive solution by constructing the Lyapunov function. The adequate conditions for the solution’s stationary distribution were described. Last but not least, the numerical simulation demonstrated that reversion rates and noise intensity influenced the disease and that there was a stationary distribution. It was concluded that the solution tends more toward the stationary distribution, the greater the reversion rates and the smaller the noise.

## 1. Introduction

The hepatitis B virus is the cause of the potentially fatal liver infection known as hepatitis B. According to the World Health Organization, we knew that the first case of acute hepatitis of unknown cause was reported in the UK on 15 April 2022. Two hundred twenty-eight children in at least 20 countries had developed liver disease by 5 May [1]. They estimated that 296 million people were lived with chronic hepatitis B infection in 2019, with about 820,000 deaths [2]. Notwithstanding the accessibility of a profoundly viable immunization, around 1.5 million individuals are recently contaminated yearly [2]. Based on the above analysis, we understood that HBV still threatens human public health. Therefore, it is important to investigate the hepatitis B virus’s dynamic behavior.

Mathematical models are regarded as an efficient method when it comes to comprehending how HBV is transmitted. In the meantime, much research has been done on the HBV infection model’s dynamic behavior [3–12]. For example, Din and Li [6] built a stochastic HBV model with Markov switching and white noise, and verified the theorem results using Runge-Kutta method. White noise plays an important role in infection control, according to reference [8] which looked at the effect of delay on HBV recurrence and reinfection. Rihan and Alsakaj looked into how a stochastic HBV model affected the persistence of the disease and the possibility of its extinction. Ge et al. [11] solved the Foker-Planck equation. In addition, the probability density function of a stochastic HBV model close to a singular local quasi-equilibrium was expressed specifically. The theoretical results are verified by numerical simulation. They are consistent with the HBV epidemic data in China.

We noted that the transmission of the hepatitis B virus is related to random environmental factors and the spatial location of the virus and cells [13–17]. In [13, 14], using the following model to investigate HBV’s dynamics:

$$\left\{\begin{array}{c} \frac{\partial u_{1}}{\partial t}=\lambda \left(x\right)-a\left(x\right)u_{1}-\beta \left(x\right)u_{1}u_{3}\;x\in \Omega ,t>0\\ \frac{\partial u_{2}}{\partial t}=\beta \left(x\right)u_{1}u_{3}-b\left(x\right)u_{2}\;x\in \Omega ,t>0\\ \frac{\partial u_{3}}{\partial t}=d△u_{3}+k\left(x\right)u_{2}-m\left(x\right)u_{3}\;x\in \Omega ,t>0, \end{array}\right.$$ (1)

where *u*_1_(*x*, *t*), *u*_2_(*x*, *t*) and *u*_3_(*x*, *t*) represent the concentration of uninfected cells, infected cells and virus, at location x and time t. λ(*x*) represents the production rate of uninfected cells. *a*(*x*) is the death rate of uninfected cells. Uninfected cells become infected cells at rate *β*(*x*)*u*_1_*u*_3_. Infected cells are produced at rate *β*(*x*)*u*_1_*u*_3_. *b*(*x*) is the death rate of infected cells. *k*(*x*) is virus production rate. *m*(*x*) is the death rate of viruses. Wu and Zou [16], in contrast to references [13, 14], focused on the diffusion of cells rather than viruses. Issa et al. [17] did not consider the spatial heterogeneity of coefficients but did consider the diffusion of viruses and cells. However, Allen [18] compared the difference between the Gaussian white noise process and the mean-reverting Ornstein-Uhlenbeck processes. The result showed that the mean-reverting Ornstein-Uhlenbeck process has better characteristics than white noise, which can describe the environmental change in biological systems well and be closer to reality theoretically and biologically. Meanwhile, the mean-reverting process is continuous, non-negative, practical and asymptotic distribution. Our simulation results also showed that as the reversion rate increases, the solution of the model is closer to the asymptotic distribution. This strategy has been generally utilized in epidemiology [19–21] and the financial economy [22, 23].

The following are the primary goals of this study: (1) By introducing cell diffusion and the mean-reverting Ornstein-Uhlenbeck process, we built the reaction-diffusion model of HBV infection. (2) The existence and uniqueness of the solution of the model and the stability of the model are proved. (3) The numerical simulation demonstrated the stationary distribution’s existence and the disease’s influence on reversion rates and noise intensity. It was concluded that the solution tends more toward the stationary distribution, the higher the reversion rate and the lower the noise.

The article’s structure is as follows: In Section 2, the mean-reverting Ornstein-Uhlenbeck process was incorporated into the diffusion HBV infection model. In Section 3, we proved the existence and uniqueness of the solution. Then, sufficient conditions are given for the diffusion HBV infection model. Numerical simulation is provided in Section 4 to demonstrate the theoretical findings. The conclusion is made in Section 5.

## 2. Model

We consider the following model:

$$\left\{\begin{array}{c} \frac{\partial u_{1}}{\partial t}=d_{1}△u_{1}+\lambda \left(x\right)-a\left(x\right)u_{1}-\beta \left(x\right)u_{1}u_{3},\;\;\;\;\;\;x\in \Omega ,t>0,\\ \frac{\partial u_{2}}{\partial t}=d_{2}△u_{2}+\beta \left(x\right)u_{1}u_{3}-b\left(x\right)u_{2},\;\;\;\;\;\;\;\;\;\;\;\;\;\;\;\;\;x\in \Omega ,t>0,\\ \frac{\partial u_{3}}{\partial t}=d_{3}△u_{3}+k\left(x\right)u_{2}-m\left(x\right)u_{3},\;\;\;\;\;\;\;\;\;\;\;\;\;\;\;\;\;\;\;\;x\in \Omega ,t>0, \end{array}\right.$$ (2)

with boundary condition

$$\begin{array}{c} \frac{\partial u_{1}}{\partial n}=\frac{\partial u_{2}}{\partial n}=\frac{\partial u_{3}}{\partial n}=0,\;x\in \partial \Omega ,\;t>0, \end{array}$$ (3)

and initial condition

$$\begin{array}{c} u_{1}\left(x,0\right)=u_{10}\left(x\right),u_{2}\left(x,0\right)=u_{20}\left(x\right),u_{3}\left(x,0\right)=u_{30}\left(x\right),x\in \Omega . \end{array}$$ (4)

The effects of a random environment are not considered in the above model. Furthermore, we introduce the mean-reverting Ornstein-Uhlenbeck process, which has the following form:

$$\left\{\begin{array}{c} da\left(x,t\right)=\vartheta_{1}\left(a_{e}-a\left(t\right)\right)dt+\varepsilon_{1}dB_{1}\left(t\right),\\ db\left(x,t\right)=\vartheta_{2}\left(b_{e}-b\left(t\right)\right)dt+\varepsilon_{2}dB_{2}\left(t\right),\\ dm\left(x,t\right)=\vartheta_{3}\left(m_{e}-m\left(t\right)\right)dt+\varepsilon_{3}dB_{3}\left(t\right), \end{array}\right.$$ (5)

where *ϑ*_*i*_, *ε*_*i*_ and *B*_*i*_(*t*), (*i* = 1, 2, 3) represent the reversion rates, noise intensity, are Brownian motion, respectively.

The stochastic integral format for the arithmetic Ornstein-Uhlenbeck process (5) enables us to obtain the following explicit form solution:

$$\left\{\begin{array}{c} a\left(t\right)=a_{e}+\left(a_{0}-a_{e}\right)e^{-\vartheta_{1}t}+\varepsilon_{1}\int_{0}^{t}e^{-\vartheta_{1}\left(t-s\right)}dB_{1}\left(s\right),\\ b\left(t\right)=b_{e}+\left(b_{0}-b_{e}\right)e^{-\vartheta_{2}t}+\varepsilon_{2}\int_{0}^{t}e^{-\vartheta_{2}\left(t-s\right)}dB_{2}\left(s\right),\\ m\left(t\right)=m_{e}+\left(m_{0}-m_{e}\right)e^{-\vartheta_{3}t}+\varepsilon_{3}\int_{0}^{t}e^{-\vartheta_{3}\left(t-s\right)}dB_{3}\left(s\right). \end{array}\right.$$ (6)

By [20], Eq (6) can be almost surely (a.s.) rewritten as:

$$\left\{\begin{array}{c} a\left(t\right)=a_{e}+\left(a_{0}-a_{e}\right)e^{-\vartheta_{1}t}+\xi_{1}\left(t\right)\frac{dB_{1}\left(t\right)}{dt},\\ b\left(t\right)=b_{e}+\left(b_{0}-b_{e}\right)e^{-\vartheta_{2}t}+\xi_{2}\left(t\right)\frac{dB_{2}\left(t\right)}{dt},\\ m\left(t\right)=m_{e}+\left(m_{0}-m_{e}\right)e^{-\vartheta_{3}t}+\xi_{3}\left(t\right)\frac{dB_{3}\left(t\right)}{dt}, \end{array}\right.$$ (7)

where *a*_0_ ≔ *a*(0) > 0, *b*_0_ ≔ *b*(0) > 0, *m*_0_ ≔ *m*(0) > 0, $\xi_{i}=\frac{\varepsilon_{i}}{2\vartheta_{i}}\sqrt{1-e^{-2\vartheta_{i}t}},\left(i=1,2,3\right)$. Substituting (7) into system (2) implies the following stochastic system

$$\left\{\begin{array}{c} du_{1}=\left[d_{1}△u_{1}+\lambda \left(x\right)-a_{e}u_{1}-\left(a_{0}-a_{e}\right)e^{-\vartheta_{1}\left(t\right)}u_{1}-\beta \left(x\right)u_{1}u_{3}\right]dt-\xi_{1}\left(t\right)u_{1}dB_{1}\left(t\right),\\ du_{2}=\left[d_{2}△u_{2}+\beta \left(x\right)u_{1}u_{3}-b_{e}u_{2}-\left(b_{0}-b_{e}\right)e^{-\vartheta_{2}t}u_{2}\right]dt-\xi_{2}\left(t\right)u_{2}dB_{2}\left(t\right),\\ du_{3}=\left[d_{3}△u_{3}+k\left(x\right)u_{2}-m_{e}u_{3}-\left(m_{{}_{0}}-m_{e}\right)e^{-\vartheta_{3}t}u_{3}\right]dt-\xi_{3}\left(t\right)u_{3}dB_{3}\left(t\right), \end{array}\right.$$ (8)

with boundary condition

$$\begin{array}{c} \frac{\partial u_{1}}{\partial n}=\frac{\partial u_{2}}{\partial n}=\frac{\partial u_{3}}{\partial n}=0,x\in \partial \Omega ,t>0, \end{array}$$

and initial condition

$$\begin{array}{c} u_{1}\left(x,0\right)=u_{10}\left(x\right),u_{2}\left(x,0\right)=u_{20}\left(x\right),u_{3}\left(x,0\right)=u_{30}\left(x\right),x\in \Omega . \end{array}$$

Let *B* be a linear operator defined by

$$\begin{array}{c} B(\begin{array}{c} u_{1}\\ u_{2}\\ u_{3} \end{array})=(\begin{array}{c} d_{1}△u_{1}\\ d_{2}△u_{2}\\ d_{3}△u_{3} \end{array}). \end{array}$$ (9)

Then, we define a nonlinear operator *C* by

$$\begin{array}{c} C(\begin{array}{c} u_{1}\\ u_{2}\\ u_{3} \end{array})=(\begin{array}{c} \lambda \left(x\right)-a_{e}u_{1}-\left(a_{0}-a_{e}\right)e^{-\vartheta_{1}\left(t\right)}u_{1}-\beta \left(x\right)u_{1}u_{3}-\xi_{1}\left(t\right)u_{1}{\dot{B}}_{1}\left(t\right)\\ \beta \left(x\right)u_{1}u_{3}-b_{e}u_{2}-\left(b_{0}-b_{e}\right)e^{-\vartheta_{2}t}u_{2}-\xi_{2}\left(t\right)u_{2}{\dot{B}}_{2}\left(t\right)\\ k\left(x\right)u_{2}-m_{e}u_{3}-\left(m_{{}_{0}}-m_{e}\right)e^{-\vartheta_{3}t}u_{3}-\xi_{3}\left(t\right)u_{3}{\dot{B}}_{3}\left(t\right) \end{array}). \end{array}$$ (10)

Let $W\left(t\right)={\left(u_{1}\left(x,t\right),u_{2}\left(x,t\right),u_{3}\left(x,t\right)\right)}^{T}$, together with Eqs (9) and (10), system (8) has been rewritten as the following abstract Cauchy problem

$$\begin{array}{c} \frac{d}{dt}W\left(t\right)=BW\left(t\right)+CW\left(t\right). \end{array}$$ (11)

## 3. Main result

### 3.1. Existence and unique of solution

Let $\left(\Omega ,F,{\left\{F_{t}\right\}}_{t\geq 0},P\right)$ be a complete probability space with a filtration ${\left\{F_{t}\right\}}_{t\geq 0}$, and *B*_*i*_(*t*), (*i* = 1, 2, 3) defined on $\left(\Omega ,F,{\left\{F_{t}\right\}}_{t\geq 0},P\right)$, $R_{+}^{3}=\left(x_{1},x_{2},x_{3}\right)\in R^{3},x_{i}>0$, (*i* = 1, 2, 3). Next, we introduce a lemma that gives a criterion for the existence of an ergodic stationary distribution to system (8).

Notation

$$\begin{array}{c} \bar{g}=\underset{t\to \infty }{\text{sup}}\;g\left(t\right),\underline{g}=\underset{t\to \infty }{\text{inf}}\;g\left(t\right), \end{array}$$ (12)

here, *g*(*t*)is a continuous bounded function.

**Lemma 3.1**. *For any initial data* (*u*_10_, *u*_20_, *u*_30_), *the solution u*(*x*, *t*) = (*u*_1_(*x*, *t*), *u*_2_(*x*, *t*), *u*_3_(*x*, *t*)) *of system* (8), *satisfies that*

$$\begin{array}{c} \text{lim}\;\underset{t\to \infty }{\text{sup}}\left(E\right\|u_{1}\left(x,t\right)\|+E\|u_{2}\left(x,t\right)\|+E\|u_{3}\left(x,t\right)\left\|\right)<M_{1}, \end{array}$$

*where M*_1_ *is a positive constant*.

*Proof*. Let

$$\begin{array}{c} N\left(t\right)=\int_{\Omega }E\left[ku_{1}\left(x,t\right)+ku_{2}\left(x,t\right)+b_{e}u_{3}\left(x,t\right)\right]dx, \end{array}$$

by (8), we have

$$\begin{array}{c} \begin{array}{cc} \frac{dN(t)}{dt} & =\int_{\Omega }E\left[k\frac{\partial }{\partial t}u_{1}\left(x,t\right)+k\frac{\partial }{\partial t}u_{2}\left(x,t\right)+b_{e}\frac{\partial }{\partial t}u_{3}\left(x,t\right)\right]dx\\ & =\int_{\Omega }E[kd_{1}△u_{1}\left(x,t\right)+k\lambda -ka_{e}u_{1}\left(x,t\right)-k\left(a_{0}-a_{e}\right)e^{-\vartheta_{1}\left(t\right)}u_{1}\left(x,t\right)-k\beta \left(x\right)u_{1}\left(x,t\right)u_{3}\left(x,t\right)\\ & -k\xi_{1}\left(t\right)u_{1}\left(x,t\right){\dot{B}}_{1}\left(t\right)+kd_{2}△u_{2}\left(x,t\right)+k\beta \left(x\right)u_{1}\left(x,t\right)u_{3}\left(x,t\right)-kb_{e}u_{2}\left(x,t\right)-k\left(b_{0}-b_{e}\right)e^{-\vartheta_{2}t}u_{2}\left(x,t\right)\\ & -k\xi_{2}\left(t\right)u_{2}\left(x,t\right){\dot{B}}_{2}\left(t\right)+b_{e}d_{3}△u_{3}\left(x,t\right)+b_{e}k\left(x\right)u_{2}\left(x,t\right)-b_{e}m_{e}u_{3}\left(x,t\right)-b_{e}\left(m_{{}_{0}}-m_{e}\right)e^{-\vartheta_{3}t}u_{3}\left(x,t\right)\\ & -b_{e}\xi_{3}\left(t\right)u_{3}\left(x,t\right){\dot{B}}_{3}\left(t\right)]\\ & \leq kd_{1}\int_{\Omega }E\left(△u_{1}\left(x,t\right)\right)dx+kd_{2}\int_{\Omega }E\left(△u_{2}\left(x,t\right)\right)dx+b_{e}d_{3}\int_{\Omega }E\left(△u_{3}\left(x,t\right)\right)\\ & +\int_{\Omega }E\left(k\lambda -ka_{e}\left(1-e^{-\vartheta_{1}t}\right)u_{1}\left(x,t\right)-kb_{e}\left(1-e^{-\vartheta_{2}t}\right)u_{2}\left(x,t\right)-b_{e}m_{e}\left(1-e^{-\vartheta_{3}t}\right)u_{3}\left(x,t\right)\right)dx\\ & \leq kd_{1}\int_{\partial \Omega }E\left(\frac{\partial }{\partial n}u_{1}\left(x,t\right)\right)dx+kd_{2}\int_{\partial \Omega }E\left(\frac{\partial }{\partial n}u_{2}\left(x,t\right)\right)dx+b_{e}d_{3}\int_{\partial \Omega }E\left(\frac{\partial }{\partial n}u_{3}\left(x,t\right)\right)dx\\ & +\int_{\Omega }E\left(k\lambda -A\left(ku_{1}\left(x,t\right)+ku_{2}\left(x,t\right)+b_{e}m_{e}u_{3}\left(x,t\right)\right)\right)dx\\ & =k\lambda |\Omega |-AN(t), \end{array} \end{array}$$

where |Ω| denotes the volume of Ω, $A=min\{a_{e}(1-e^{-\vartheta_{1}t}),b_{e}(1-e^{-\vartheta_{2}t}),m_{e}(1-e^{-\vartheta_{3}t})\}$. This implies that

$$\begin{array}{c} \underset{t\to +\infty }{\text{lim}}\;N\left(t\right)\leq \frac{k\lambda |\Omega |}{A}≔M_{1}. \end{array}$$

**Remark 1** Lemma 3.1 means that the solution is boundness for system (8).

Furthermore, we prove the existence and unique of solution.

**Theorem 3.2** *For any initial data* (*u*_10_, *u*_20_, *u*_30_) > 0, *there exists a unique solution* (*u*_1_(*x*, *t*), *u*_2_(*x*, *t*), *u*_3_(*x*, *t*)) > 0 *of system* (8) *for t* > 0 *on* Ω.

*Proof*. Since the coefficients of system (8) satisfy the local Lipschitz condition, there is a unique local solution on *t* ∈ [0, *τ*_*e*_), where *τ*_*e*_ is the explosion time Let *l*_0_ > 0 be sufficiently large for

$$\begin{array}{c} \frac{1}{l_{0}}\leq \;\underset{0<t<\tau_{e}}{\text{min}}\;\left|W\left(t\right)\right|\leq \;\underset{0<t<\tau_{e}}{\text{max}}\;\left|W\left(t\right)\right|\leq l_{0}. \end{array}$$

For each integer *l* > *l*_0_, define the stopping time

$$\begin{array}{c} \tau_{l}=inf\left\{t\in \left[0,\tau_{e}\right]:\text{min}\left(u_{1},u_{2},u_{3}\right)\leq \frac{1}{l}\;or\;\text{max}\left(u_{1},u_{2},u_{3}\right)\geq l\right\}. \end{array}$$

Let *inf* ∅ = ∞ (∅ represents the empty set). *τ*_*l*_ is increasing as *l* → ∞. Let *τ*_∞_ = lim_*l*→∞_ *τ*_*l*_, then *τ*_∞_ < *τ*_*e*_ a.s. In the following, we need to show *τ*_∞_ = ∞ a.s. Therefore, according to Itô’s formula, we have

$$\begin{array}{c} \begin{array}{cc} d( & \|u_{1}{\left(x,t\right)\|}^{2}+\|u_{2}{\left(x,t\right)\|}^{2}+\|u_{3}\left(x,t\right){\|}^{2})\\ & =\{2\left\langle u_{1}\left(x,t\right),d_{1}△u_{1}+\lambda \left(x\right)-a_{e}u_{1}-\left(a_{0}-a_{e}\right)e^{-\vartheta_{1}\left(t\right)}u_{1}-\beta \left(x\right)u_{1}u_{3}\right\rangle \\ & +2\langle u_{2}\left(x,t\right),d_{2}△u_{2}+\beta \left(x\right)u_{1}u_{3}-b_{e}u_{2}-\left(b_{0}-b_{e}\right)e^{-\vartheta_{2}t}u_{2}\rangle \\ & +2\left\langle u_{3}\left(x,t\right),d_{3}△u_{3}+k\left(x\right)u_{2}-m_{e}u_{3}-\left(m_{{}_{0}}-m_{e}\right)e^{-\vartheta_{3}t}u_{3}\right\rangle +\xi_{1}^{2}\left(t\right){\left\|u_{1}\left(x,t\right)\right\|}^{2}\\ & +\xi_{2}^{2}\left(t\right)\|u_{2}{\left(x,t\right)\|}^{2}+\varepsilon_{3}^{2}\left(t\right)\|u_{3}\left(x,t\right){\|}^{2}\}dt+2\left\langle u_{1}\left(x,t\right),-\xi_{1}u_{1}\left(x,t\right)dB_{1}\left(t\right)\right\rangle \\ & +2\left\langle u_{2}\left(x,t\right),-\xi_{2}u_{2}\left(x,t\right)dB_{2}\left(t\right)\right\rangle +2\left\langle u_{3}\left(x,t\right),-\xi_{3}u_{3}\left(x,t\right)dB_{3}\left(t\right)\right\rangle . \end{array} \end{array}$$ (13)

Now, let *l* > *l*_0_ and *T* > 0, we can integrate both sides of (13) from 0 to *τ*_*l*_ ∧ *T* and then take the expectations to get

$$\begin{array}{c} \begin{array}{cc} & E[\|u_{1}\left(x,\tau_{l}\wedge T\right){\|}^{2}+\|u_{2}\left(x,\tau_{l}\wedge T\right){\|}^{2}+\|u_{3}\left(x,\tau_{l}\wedge T\right){\|}^{2}]-(\|u_{10}{\|}^{2}+\|u_{20}{\|}^{2}+\|u_{30}{\|}^{2})\\ & =E\int_{0}^{\tau_{l}\wedge T}\{-2d_{1}\|\nabla u_{1}{\left(x,s\right)\|}^{2}+2\left\langle u_{1}\left(x,s\right),\lambda \right\rangle -2a_{e}\|u_{1}{\left(x,s\right)\|}^{2}-2\left(a_{0}-a_{e}\right)e^{-\vartheta_{1}t}{\left\|u_{1}\left(x,s\right)\right\|}^{2}\\ & -2\left\langle u_{1}\left(x,s\right),\beta u_{1}\left(x,s\right)u_{3}\left(x,s\right)\right\rangle -2d_{2}\|\nabla u_{2}{\left(x,s\right)\|}^{2}+2\left\langle u_{2}\left(x,s\right),\beta u_{1}\left(x,s\right)u_{3}\left(x,s\right)\right\rangle -2b_{e}{\left\|u_{2}\left(x,s\right)\right\|}^{2}\\ & -2\left(b_{0}-b_{e}\right)e^{-\vartheta_{2}t}\|u_{2}\left(x,s\right){\|}^{2}-2d_{3}\|\nabla u_{3}\left(x,s\right){\|}^{2}+2\left\langle u_{3}\left(x,s\right),ku_{2}\left(x,s\right)\right\rangle -2m_{e}{\left\|u_{3}\left(x,s\right)\right\|}^{2}-\\ & 2\left(m_{0}-m_{e}\right)e^{-\vartheta_{3}t}\|u_{3}\left(x,s\right){\|}^{2}+\xi_{1}{\left(s\right)}^{2}\|u_{1}\left(x,s\right){\|}^{2}+\xi_{2}{\left(s\right)}^{2}u_{2}\left(x,s\right)+\xi_{3}{\left(s\right)}^{2}u_{3}\left(x,s\right)\}ds\\ & \leq E\int_{0}^{\tau_{l}\wedge T}\{2\left\langle u_{1}\left(x,s\right),\lambda \right\rangle +2\left\langle u_{2}\left(x,s\right),\beta u_{1}\left(x,s\right)u_{3}\left(x,s\right)\right\rangle +2\left\langle u_{3}\left(x,s\right),ku_{2}\left(x,s\right)\right\rangle +\xi_{1}^{2}\left(s\right){\left\|u_{1}\left(x,s\right)\right\|}^{2}+\\ & \xi_{2}^{2}\left(s\right)u_{2}\left(x,s\right)+\xi_{3}^{2}\left(s\right)u_{3}\left(x,s\right)\}ds. \end{array} \end{array}$$

Then according to Lemma 3.1 and fundamental inequality, we have

$$\begin{array}{c} \begin{array}{cc} E & \left[\|u_{1}\left(x,\tau_{l}\wedge T\right){\|}^{2}+\|u_{2}\left(x,\tau_{l}\wedge T\right){\|}^{2}+\|u_{3}\left(x,\tau_{l}\wedge T\right){\|}^{2}\right]\\ & \leq \left(\right\|u_{10}{\|}^{2}+\|u_{20}{\|}^{2}+\|u_{30}{\|}^{2})+E\int_{0}^{\tau_{l}\wedge T}\left\{\right\|u_{1}{\left(x,s\right)\|}^{2}+{\bar{\lambda }}^{2}+\|u_{2}{\left(x,s\right)\|}^{2}+\beta^{2}M_{1}^{2}{\left\|u_{3}\left(x,s\right)\right\|}^{2}\\ & +\|u_{3}{\left(x,s\right)\|}^{2}+k^{2}\|u_{2}{\left(x,s\right)\|}^{2}+\xi_{1}^{2}\left(s\right)\|u_{1}\left(x,s\right){\|}^{2}+\xi_{2}^{2}\left(s\right)u_{2}\left(x,s\right)+\xi_{3}^{2}\left(s\right)u_{3}\left(x,s\right)\}ds\\ & \leq M_{2}+M_{3}E\int_{0}^{\tau_{l}\wedge T}\left\{\right\|u_{1}{\left(x,s\right)\|}^{2}+\|u_{2}{\left(x,s\right)\|}^{2}+\|u_{3}\left(x,s\right){\|}^{2}\}ds, \end{array} \end{array}$$

where

$$\begin{array}{c} \begin{array}{cc} & M_{2}=\|u_{10}{\|}^{2}+\|u_{20}{\|}^{2}+{\left\|u_{30}\right\|}^{2}+{\bar{\lambda }}^{2}\tau_{l},\\ & M_{3}=max\left\{\left(1+\xi_{1}^{2}\left(s\right)\right),\left(1+k^{2}+\xi_{2}^{2}\left(s\right)\right),\left(1+\beta^{2}M_{1}^{2}+\xi_{3}^{2}\left(s\right)\right)\right\}. \end{array} \end{array}$$

By the Gronwall inequality, we have

$$\begin{array}{c} E[\|u_{1}\left(x,\tau_{l}\wedge T\right){\|}^{2}+\|u_{2}\left(x,\tau_{l}\wedge T\right){\|}^{2}+\|u_{3}\left(x,\tau_{l}\wedge T\right){\|}^{2}]\leq M_{2}e^{M_{3}T}. \end{array}$$ (14)

Define

$$\begin{array}{c} \lambda_{l}=\underset{\|W(t)\|>l,0<t<\infty }{\text{inf}}\left(\right\|u_{1}{\left(x,t\right)\|}^{2}+\|u_{2}{\left(x,t\right)\|}^{2}+\|u_{3}\left(x,t\right){\|}^{2}),for\;any\;l>l_{0}. \end{array}$$ (15)

Combine (14) and (15) to get

$$\begin{array}{c} \lambda_{l}P\left(\tau_{l}\leq T\right)\leq M_{2}e^{M_{3}T}, \end{array}$$

since lim_*l*→∞_ λ_*l*_ = ∞, in the above inequality, let *l* → ∞, we can get *P*(*τ*_∞_ ≤ *T*) = 0, namely,

$$\begin{array}{c} P(\tau_{l}\geq T)=1. \end{array}$$

By (14), *l* → ∞ means that

$$\begin{array}{c} E[\|u_{1}{\left(x,T\right)\|}^{2}+\|u_{2}{\left(x,T\right)\|}^{2}+\|u_{3}\left(x,T\right){\|}^{2}]\leq M_{2}e^{M_{3}T}. \end{array}$$

This proof is complete. The above theorem represents the system (8) exists a unique global solution.

**Remark 2** Theorem 3.2 represents the system (8) exists a unique global solution.

**Theorem 3.3** *With respect to the function V* = ‖*u*_1_(*x*, *t*)‖^2^ + ‖*u*_2_(*x*, *t*)‖^2^ + ‖*u*_3_(*x*, *t*)‖^2^, *we have*

$$\begin{array}{c} \underset{t\to \infty }{\text{lim}}\;\text{sup}\frac{1}{t}ln(E(\|u_{1}{\left(x,t\right)\|}^{2}+\|u_{2}{\left(x,t\right)\|}^{2}+\|u_{3}{\left(x,t\right)\|}^{2}))\leq M_{3}. \end{array}$$

*Proof*. By virtue of Eq (13), we have $V\leq -\frac{{\bar{\lambda }}^{2}}{M_{3}}+ce^{M_{3}t}$ (where *c* > 0 is a constant). Moreover, we will prove the bounded of *LV*, according to the Eq (13), we can obtain

$$\begin{array}{c} \begin{array}{cc} LV= & 2\langle u_{1}\left(x,t\right),d_{1}△u_{1}+\lambda \left(x\right)-a_{e}u_{1}-\left(a_{0}-a_{e}\right)e^{-\vartheta_{1}\left(t\right)}u_{1}-\beta \left(x\right)u_{1}u_{3}\rangle \\ & +2\langle u_{2}\left(x,t\right),d_{2}△u_{2}+\beta \left(x\right)u_{1}u_{3}-b_{e}u_{2}-\left(b_{0}-b_{e}\right)e^{-\vartheta_{2}t}u_{2}\rangle \\ & +2\langle u_{3}\left(x,t\right),d_{3}△u_{3}+k\left(x\right)u_{2}-m_{e}u_{3}-\left(m_{{}_{0}}-m_{e}\right)e^{-\vartheta_{3}t}u_{3}\rangle \\ & +\xi_{1}^{2}\left(t\right)\|u_{1}{\left(x,t\right)\|}^{2}+\xi_{2}^{2}\left(t\right)\|u_{2}{\left(x,t\right)\|}^{2}+\varepsilon_{3}^{2}\left(t\right){\left\|u_{3}\left(x,t\right)\right\|}^{2}\\ = & -2d_{1}\|\nabla u_{1}{\left(x,s\right)\|}^{2}+2\left\langle u_{1}\left(x,s\right),\lambda \right\rangle -2a_{e}\|u_{1}{\left(x,s\right)\|}^{2}-2\left(a_{0}-a_{e}\right)e^{-\vartheta_{1}t}{\left\|u_{1}\left(x,s\right)\right\|}^{2}\\ & -2\left\langle u_{1}\left(x,s\right),\beta u_{1}\left(x,s\right)u_{3}\left(x,s\right)\right\rangle -2d_{2}\|\nabla u_{2}{\left(x,s\right)\|}^{2}+2\left\langle u_{2}\left(x,s\right),\beta u_{1}\left(x,s\right)u_{3}\left(x,s\right)\right\rangle -2b_{e}{\left\|u_{2}\left(x,s\right)\right\|}^{2}\\ & -2\left(b_{0}-b_{e}\right)e^{-\vartheta_{2}t}\|u_{2}\left(x,s\right){\|}^{2}-2d_{3}\|\nabla u_{3}\left(x,s\right){\|}^{2}+2\left\langle u_{3}\left(x,s\right),ku_{2}\left(x,s\right)\right\rangle -2m_{e}{\left\|u_{3}\left(x,s\right)\right\|}^{2}-\\ & 2\left(m_{0}-m_{e}\right)e^{-\vartheta_{3}t}\|u_{3}\left(x,s\right){\|}^{2}+\xi_{1}{\left(s\right)}^{2}\|u_{1}\left(x,s\right){\|}^{2}+\xi_{2}{\left(s\right)}^{2}u_{2}\left(x,s\right)+\xi_{3}{\left(s\right)}^{2}u_{3}\left(x,s\right)\}ds\\ & \leq {\bar{\lambda }}^{2}+M_{3}\left(\right\|u_{1}{\left(x,t\right)\|}^{2}+\|u_{2}{\left(x,s\right)\|}^{2}+\|u_{3}\left(x,s\right){\|}^{2}). \end{array} \end{array}$$

For *V*, using Itô’s formula:

$$\begin{array}{c} \begin{array}{cc} EV & =\left(\right\|u_{10}{\|}^{2}+\|u_{20}{\|}^{2}+\|u_{30}{\|}^{2})+E\int_{0}^{t}LVds\\ & \leq \left(\right\|u_{10}{\|}^{2}+\|u_{20}{\|}^{2}+\|u_{30}{\|}^{2})+{\bar{\lambda }}^{2}t+M_{3}E\int_{0}^{t}\left((\right\|u_{1}{\left(x,t\right)\|}^{2}+\|u_{2}{\left(x,s\right)\|}^{2}+\|u_{3}{\left(x,s\right)\|}^{2}))ds\\ & \leq \left(\right\|u_{10}{\|}^{2}+\|u_{20}{\|}^{2}+\|u_{30}{\|}^{2})+{\bar{\lambda }}^{2}t+M_{3}E\int_{0}^{t}\left(-\frac{{\bar{\lambda }}^{2}}{M_{3}}+ce^{M_{3}t}\right)ds. \end{array} \end{array}$$

According to the arbitrariness of *c*, we have

$$\begin{array}{c} EV\leq \frac{ce^{M_{3}t}}{M_{3}}. \end{array}$$

The result of the theorem can be obtained.

**Remark 3** Theorem 3.3 denotes the square exponent stability of the Lyapunov function.

**Theorem 3.4**. *If E*(‖*u*_10_‖^2^ + ‖*u*_20_‖^2^ + ‖*u*_30_‖^2^) ≤ *Z*_1_, *we have*

$$\begin{array}{c} E(\|u_{1}{\left(x,t\right)\|}^{2}+\|u_{2}{\left(x,t\right)\|}^{2}+\|u_{3}\left(x,t\right){\|}^{2})\leq Z_{2},\;\;t\in \left[0,T\right], \end{array}$$

*where Z*_1_, *Z*_2_, *T are positive real numbers. Then system* (8) *is finite-time stable*.

*Proof*. According to Theorem 3.2, we can obtain the proof of the theorem.

**Remark 4** Theorem 3.4 denotes the model is finite-time stable.

Next, we prove the stationary distribution of the solution for system (8).

### 3.2. Stationary distribution of solution

First, we introduce the follow theorem.

**Theorem 3.5** *For any κ* > 0, *we have*

$$\begin{array}{c} E(\underset{0\leq t\leq T}{\text{sup}}\|u_{1}{\left(x,t\right)\|}^{k}+\underset{0\leq t\leq T}{\text{sup}}\|u_{1}{\left(x,t\right)\|}^{k}+\underset{0\leq t\leq T}{\text{sup}}\|u_{1}\left(x,t\right){\|}^{k})\leq M_{\kappa }, \end{array}$$

*where M*_*κ*_ *is a constant that depends only on κ*.

*Proof*. First, we consider *κ* > 1, By applying the Itô’s formula, we have

$$\begin{array}{c} \begin{array}{cc} & \|u_{1}{\left(x,s\right)\|}^{\kappa }+\|u_{2}{\left(x,s\right)\|}^{\kappa }+\|u_{3}{\left(x,s\right)\|}^{\kappa }-\|u_{10}{\|}^{\kappa }-\|u_{20}{\|}^{\kappa }-{\left\|u_{30}\right\|}^{\kappa }\\ = & \int_{0}^{t}\left\{\kappa \right\|u_{1}{\left(x,s\right)\|}^{\kappa -2}\left\langle u_{1}\left(x,s\right),d_{1}△u_{1}+\lambda \left(x\right)-a_{e}u_{1}-\left(a_{0}-a_{e}\right)e^{-\vartheta_{1}\left(t\right)}u_{1}-\beta \left(x\right)u_{1}u_{3}\right\rangle \\ & +\kappa \|u_{2}{\left(x,s\right)\|}^{\kappa -2}\left\langle u_{2}\left(x,s\right),d_{2}△u_{2}+\beta \left(x\right)u_{1}u_{3}-b_{e}u_{2}-\left(b_{0}-b_{e}\right)e^{-\vartheta_{2}t}u_{2}\right\rangle +\kappa {\left\|u_{3}\left(x,s\right)\right\|}^{\kappa -2}\\ & \left\langle u_{3}\left(x,s\right),d_{3}△u_{3}+k\left(x\right)u_{2}-m_{e}u_{3}-\left(m_{{}_{0}}-m_{e}\right)e^{-\vartheta_{3}t}u_{3}\right\rangle +\frac{1}{2}\kappa \left(\kappa -1\right)\xi_{1}^{2}\left(s\right){\left\|u_{1}\left(x,s\right)\right\|}^{2}\\ & +\frac{1}{2}\kappa \left(\kappa -1\right)\xi_{2}^{2}\left(s\right)\|u_{2}{\left(x,s\right)}^{2}\|+\frac{1}{2}\kappa \left(\kappa -1\right)\xi_{3}^{2}\left(s\right)\|u_{3}{\left(x,s\right)}^{2}\|\}ds-\int_{0}^{t}\kappa \xi_{1}\left(s\right){\left\|u_{1}\left(x,s\right)\right\|}^{\kappa }dB_{1}\left(s\right)\\ & -\int_{0}^{t}\kappa \xi_{2}\left(s\right)\|u_{{}_{2}}{\left(x,s\right)\|}^{\kappa }dB_{2}\left(s\right)-\int_{0}^{t}\kappa \xi_{3}\left(s\right){\left\|u_{3}\left(x,s\right)\right\|}^{\kappa }dB_{1}\left(s\right). \end{array} \end{array}$$

Next, we take the *sup*(⋅) and expectation of the above equation

$$\begin{array}{c} \begin{array}{cc} & E\underset{0\leq t\leq T}{\text{sup}}\left\{\right\|u_{1}{\left(x,s\right)\|}^{\kappa }+\|u_{2}{\left(x,s\right)\|}^{\kappa }+\|u_{3}\left(x,s\right){\|}^{\kappa }\}\\ & \leq E\underset{0\leq t\leq T}{\text{sup}}\left(\right\|u_{10}{\|}^{\kappa }+\|u_{20}{\|}^{\kappa }+\|u_{30}{\|}^{\kappa })+E\underset{0\leq t\leq T}{\text{sup}}\int_{0}^{t}\{-d_{1}\kappa \|u_{1}{\left(x,s\right)\|}^{\kappa -2}{\left\|\nabla u_{1}\left(x,s\right)\right\|}^{2}\\ & +\kappa \lambda \|u_{1}{\left(x,s\right)\|}^{\kappa -1}-\kappa a_{e}\|u_{1}{\left(x,s\right)\|}^{\kappa }-\kappa \left(a_{0}-a_{e}\right)e^{-\vartheta_{1}t}\|u_{1}\left(x,s\right){\|}^{\kappa }-\kappa \beta {\left\|u_{1}\left(x,s\right)\right\|}^{2}\\ & \left\langle u_{1}\left(x,s\right),u_{1}\left(x,s\right)u_{3}\left(x,s\right)\right\rangle -d_{2}\kappa \|u_{2}{\left(x,s\right)\|}^{\kappa -2}\|\nabla u_{2}{\left(x,s\right)\|}^{2}-\kappa b_{e}{\left\|u_{2}\left(x,s\right)\right\|}^{2}\\ & +\kappa \beta \|u_{2}{\left(x,s\right)\|}^{\kappa -2}\left\langle u_{2}\left(x,s\right),u_{1}\left(x,s\right)u_{3}\left(x,s\right)\right\rangle -\kappa \left(b_{0}-b_{e}\right)e^{-\vartheta_{3}t}\|u_{2}\left(x,s\right){\|}^{2}-d_{3}\kappa {\left\|u_{3}\left(x,s\right)\right\|}^{\kappa -2}\\ & \|\nabla u_{1}{\left(x,s\right)\|}^{2}+\kappa k\|u_{3}{\left(x,s\right)\|}^{\kappa -2}\left\langle u_{3}\left(x,s\right),u_{2}\left(x,s\right)\right\rangle -\kappa m_{e}{\left\|u_{3}\left(x,s\right)\right\|}^{\kappa }-\kappa \left(m_{0}-m_{e}\right)e^{-\vartheta_{3}t}\\ & \|u_{3}{\left(x,s\right)\|}^{\kappa }+\frac{1}{2}\kappa \left(\kappa -1\right)\xi_{1}^{2}\left(s\right)\|u_{1}{\left(x,s\right)\|}^{2}+\frac{1}{2}\kappa \left(\kappa -1\right)\xi_{2}^{2}\left(s\right){\left\|u_{2}\left(x,s\right)\right\|}^{2}+\frac{1}{2}\kappa \left(\kappa -1\right)\xi_{3}^{2}\left(s\right)\\ & \|u_{3}{\left(x,s\right)\|}^{2}\}ds-E\underset{0\leq t\leq T}{\text{sup}}\int_{0}^{t}\kappa \xi_{1}\left(s\right)\|u_{1}{\left(x,s\right)\|}^{\kappa }dB_{1}\left(s\right)-E\underset{0\leq t\leq T}{\text{sup}}\int_{0}^{t}\kappa \xi_{2}\left(s\right){\left\|u_{{}_{2}}\left(x,s\right)\right\|}^{\kappa }dB_{2}\left(s\right)\\ & -E\underset{0\leq t\leq T}{\text{sup}}\int_{0}^{t}\kappa \xi_{3}\left(s\right){\left\|u_{3}\left(x,s\right)\right\|}^{\kappa }dB_{3}\left(s\right)\\ & \leq E\underset{0\leq t\leq T}{\text{sup}}\left(\right\|u_{10}{\|}^{\kappa }+\|u_{20}{\|}^{\kappa }+\|u_{30}{\|}^{\kappa })+E\underset{0\leq t\leq T}{\text{sup}}\int_{0}^{t}\left\{\kappa \lambda \right\|u_{1}{\left(x,s\right)\|}^{\kappa -1}+\kappa \beta {\left\|u_{2}\left(x,s\right)\right\|}^{\kappa -2}\\ & \left\langle u_{2}\left(x,s\right),u_{1}\left(x,s\right)u_{3}\left(x,s\right)\right\rangle +\kappa k{\left\|u_{3}\left(x,s\right)\right\|}^{\kappa -2}\left\langle u_{3}\left(x,s\right),u_{2}\left(x,s\right)\right\rangle +\frac{1}{2}\kappa \left(\kappa -1\right) \end{array} \end{array}$$

$$\begin{array}{c} \begin{array}{cc} & \xi_{1}^{2}\left(s\right)\|u_{1}{\left(x,s\right)\|}^{2}+\frac{1}{2}\kappa \left(\kappa -1\right)\xi_{2}^{2}\left(s\right)\|u_{2}{\left(x,s\right)}^{2}\|+\frac{1}{2}\kappa \left(\kappa -1\right)\xi_{3}^{2}\left(s\right)\|u_{3}{\left(x,s\right)}^{2}\left\|\right\}ds\\ & -E\underset{0\leq t\leq T}{\text{sup}}\int_{0}^{t}\kappa \xi_{1}\left(s\right)\|u_{1}{\left(x,s\right)\|}^{\kappa }dB_{1}\left(s\right)-E\underset{0\leq t\leq T}{\text{sup}}\int_{0}^{t}\kappa \xi_{2}\left(s\right){\left\|u_{{}_{2}}\left(x,s\right)\right\|}^{\kappa }dB_{2}\left(s\right)\\ & -E\underset{0\leq t\leq T}{\text{sup}}\int_{0}^{t}\kappa \xi_{3}\left(s\right){\left\|u_{3}\left(x,s\right)\right\|}^{\kappa }dB_{3}\left(s\right). \end{array} \end{array}$$

Using the Young inequality and Burkholder-Davis-Gundy inequality, we have

$$\begin{array}{c} \begin{array}{cc} & E\underset{0\leq t\leq T}{\text{sup}}\left\{\right\|u_{1}{\left(x,s\right)\|}^{\kappa }+\|u_{2}{\left(x,s\right)\|}^{\kappa }+\|u_{3}\left(x,s\right){\|}^{\kappa }\}\\ & \leq E(\|u_{10}{\|}^{\kappa }+\|u_{20}{\|}^{\kappa }+\|u_{30}{\|}^{\kappa })+E\underset{0\leq t\leq T}{\text{sup}}\int_{0}^{t}\{\lambda^{\kappa }+\left(\kappa -1+\frac{1}{2}\kappa \left(\kappa -1\right)\xi_{1}^{2}\right){\left\|u_{1}\left(x,s\right)\right\|}^{2}\\ & +\left(\kappa -1+k^{\kappa }+\frac{1}{2}\kappa \left(\kappa -1\right)\xi_{2}^{2}\right)\|u_{2}{\left(x,s\right)\|}^{2}+\left(\kappa -1+\beta^{\kappa }M_{1}^{\kappa }+\frac{1}{2}\kappa \left(\kappa -1\right)\xi_{3}^{2}\right)\|u_{3}\left(x,s\right){\|}^{2}\}\\ & +E\underset{0\leq t\leq T}{\text{sup}}|\int_{0}^{t}\kappa \xi_{1}\left(s\right)\|u_{1}{\left(x,s\right)\|}^{\kappa }dB_{1}\left(s\right)||+E\underset{0\leq t\leq T}{\text{sup}}|\int_{0}^{t}\kappa \xi_{2}\left(s\right)\|u_{{}_{2}}\left(x,s\right){\|}^{\kappa }dB_{2}\left(s\right)|\\ & +E\underset{0\leq t\leq T}{\text{sup}}|\int_{0}^{t}\kappa \xi_{3}\left(s\right)\|u_{3}\left(x,s\right){\|}^{\kappa }dB_{3}\left(s\right)| \end{array} \end{array}$$

$$\begin{array}{c} \begin{array}{cc} & \leq E(\|u_{10}{\|}^{\kappa }+\|u_{20}{\|}^{\kappa }+\|u_{30}{\|}^{\kappa })+\lambda^{\kappa }T+M_{4}E\underset{0\leq t\leq T}{\text{sup}}\int_{0}^{t}\left\{\right\|u_{1}{\left(x,s\right)\|}^{\kappa }+\|u_{2}{\left(x,s\right)\|}^{\kappa }+\|u_{3}\left(x,s\right){\|}^{\kappa }\}ds\\ & +E\underset{0\leq t\leq T}{\text{sup}}\|u_{1}{\left(x,s\right)\|}^{\kappa /2}(\int_{0}^{t}\kappa^{2}\xi_{1}^{2}\left(s\right)\|u_{1}{\left(x,s\right)\|}^{2}{)}^{1/2}+E\underset{0\leq t\leq T}{\text{sup}}\|u_{2}{\left(x,s\right)\|}^{\kappa /2}(\int_{0}^{t}\kappa^{2}\xi_{1}^{2}\left(s\right)\|u_{2}\left(x,s\right){{\|}^{2})}^{1/2}\\ & +E\underset{0\leq t\leq T}{\text{sup}}\|u_{3}{\left(x,s\right)\|}^{\kappa /2}(\int_{0}^{t}\kappa^{2}\xi_{1}^{2}\left(s\right)\|u_{3}\left(x,s\right){{\|}^{2})}^{1/2}\\ & \leq E(\|u_{10}{\|}^{\kappa }+\|u_{20}{\|}^{\kappa }+\|u_{30}{\|}^{\kappa })+\lambda^{\kappa }T+M_{4}E\underset{0\leq t\leq T}{\text{sup}}\int_{0}^{t}\left\{\right\|u_{1}{\left(x,s\right)\|}^{\kappa }+\|u_{2}{\left(x,s\right)\|}^{\kappa }+\|u_{3}\left(x,s\right){\|}^{\kappa }\}ds\\ & +\frac{1}{2}E\underset{0\leq t\leq T}{\text{sup}}\left(\right\|u_{1}{\left(x,s\right)\|}^{\kappa }+\|u_{2}{\left(x,s\right)\|}^{\kappa }+\|u_{3}\left(x,s\right){\|}^{\kappa })\\ & \leq 2E(\|u_{10}{\|}^{\kappa }+\|u_{20}{\|}^{\kappa }+\|u_{30}{\|}^{\kappa })+2\lambda^{\kappa }T+M_{4}E\underset{0\leq t\leq T}{\text{sup}}\int_{0}^{t}\left\{\right\|u_{1}{\left(x,s\right)\|}^{\kappa }+\|u_{2}{\left(x,s\right)\|}^{\kappa }+\|u_{3}\left(x,s\right){\|}^{\kappa }\}ds, \end{array} \end{array}$$

where

$$\begin{array}{c} M_{4}=max\left\{\kappa -1+\frac{1}{2}\kappa \left(\kappa -1\right)\xi_{1}^{2},\kappa -1+k^{\kappa }+\frac{1}{2}\kappa \left(\kappa -1\right)\xi_{2}^{2},\kappa -1+\beta^{\kappa }M_{1}^{\kappa }+\frac{1}{2}\kappa \left(\kappa -1\right)\xi_{3}^{2}\right\}. \end{array}$$

According to the Gronwall inequality, we obtained

$$\begin{array}{c} \begin{array}{cc} E & \underset{0\leq t\leq T}{\text{sup}}\left\{\right\|u_{1}{\left(x,s\right)\|}^{\kappa }+\|u_{2}{\left(x,s\right)\|}^{\kappa }+\|u_{3}\left(x,s\right){\|}^{\kappa }\}\\ & \leq \left(2E(\right\|u_{10}{\|}^{\kappa }+\|u_{20}{\|}^{\kappa }+\|u_{30}{\|}^{\kappa })+2\lambda^{\kappa }T)e^{2M_{4}T}\\ & ≔M_{\kappa }. \end{array} \end{array}$$

For 0 < *κ* < 1, based on the Cauchy-Schwartz inequality, we obtain

$$\begin{array}{c} \begin{array}{cc} E & \underset{0\leq t\leq T}{\text{sup}}\left\{\right\|u_{1}{\left(x,s\right)\|}^{\kappa }+\|u_{2}{\left(x,s\right)\|}^{\kappa }+\|u_{3}\left(x,s\right){\|}^{\kappa }\}\\ & \leq {\left(E1^{\frac{2}{2-\kappa }}\right)}^{1-\kappa /2}\{E(\underset{0\leq s\leq T}{\text{sup}}\left\{\right\|u_{1}\left(x,s\right){\|}^{\kappa }+\|u_{2}\left(x,s\right){\|}^{\kappa }+\|u_{3}\left(x,s\right){\|}^{\kappa }{{)}^{\frac{2}{\kappa }}\}}^{\frac{\kappa }{2}}\\ & ≔M_{\kappa }. \end{array} \end{array}$$

This proof is completed.

**Remark 5** Theorem 3.5 indicates that the solution of the model is *k*–moment bounded.

Next, we will give sufficient conditions for the existence and uniqueness of stationary distribution of the solution to the diffusion HBV infection model.

**Definition 3.1** [24] A stationary distribution for $W\left(x,t\right)=(u_{1}\left(x,t\right),u_{2}\left(x,t\right),u_{3}\left(x,t\right)),t\geq 0$, of system (8) is defined as a probability measure λ ∈ *P*(Ω) satisfying

$$\begin{array}{c} \lambda \left(f\right)=\lambda \left(P_{t}f\right),\;t>0, \end{array}$$

here

$$\begin{array}{c} \lambda \left(f\right)≔\int_{\Omega }f\left(\psi \right)\lambda \left(d\psi \right),P_{t}f\left(\psi \right)≔Ef\left(W\left(x,t,\psi \right)\right),f\in C_{b}\left(\Omega \right). \end{array}$$

For λ_1_, λ_2_ ∈ *P*(Ω), define a metric on *P*(Ω) by

$$\begin{array}{c} d\left(\lambda_{1},\lambda_{2}\right)=\underset{f\in A}{\text{sup}}\left|\int_{\Omega }f\left(\psi \right)\lambda_{1}\left(d\psi \right)-f\left(\varphi \right)\lambda_{2}\left(d\varphi \right)\right| \end{array}$$

where

$$\begin{array}{c} A≔\{f:\Omega \to R,|f\left(\psi \right)-f\left(\varphi \right)|\leq |\psi -\varphi {|}_{\Omega },\psi ,\varphi \in \Omega \;and\;|f\left(\cdot \right)|\leq 1\}, \end{array}$$

*P*(Ω) is complete under the metric *d*(⋅, ⋅). So, we have the following lemma

**Lemma 3.6** *For any bounded subset B* of Ω, *m* ≥ 1, *we have*

(1) $\underset{t\to \infty }{\text{lim}}\underset{\psi ,\varphi \in B}{\text{sup}}\;{E\|W\left(x,t,\psi \right)-W\left(x,t,\varphi \right)\|}_{\Omega }^{m}=0$;

(2) $\underset{t\to \infty }{\text{lim}}\;\underset{\psi \in B}{\text{sup}}E{\left\|W\left(x,t,\psi \right)\right\|}_{\Omega }^{m}<\infty$.

**Theorem 3.7** *For system* (8), *there exists a unique stationary distribution* λ ∈ *P*(Ω) *for* $W\left(x,t\right)=(u_{1}\left(x,t\right),u_{2}\left(x,t\right),u_{3}\left(x,t\right)),t\geq 0$.

*Proof*. The Theorem 3.5 is equal to condition (2) in Lemma 3.6. In order to complete proof, we only need to verify that condition (1) is valid. Next, we consider the difference of two mild solutions of system (8) with distinct initial data *ψ*, *φ* ∈ Ω

$$\begin{array}{c} e\left(x,t\right)=(\begin{array}{c} e_{1}\left(x,t,\psi ,\varphi \right)\\ e_{2}\left(x,t,\psi ,\varphi \right)\\ e3(x,t,\psi ,\varphi ) \end{array})=(\begin{array}{c} u_{1}\left(x,t,\psi \right)-u_{1}\left(x,t,\varphi \right)\\ u_{2}\left(x,t,\psi \right)-u_{2}\left(x,t,\varphi \right)\\ u_{3}\left(x,t,\psi \right)-u_{3}\left(x,t,\varphi \right) \end{array}), \end{array}$$ (16)

with ‖*e*(*x*, *t*, *ψ*, *φ*)‖^*κ*^ = ‖*e*_1_(*x*, *t*, *ψ*, *φ*)‖^*κ*^+ ‖*e*_2_(*x*, *t*, *ψ*, *φ*)‖^*κ*^+ ‖*e*_3_(*x*, *t*, *ψ*, *φ*)‖^*κ*^, by lemma 3.1 and Itô’s formula, we have

$$\begin{array}{c} \begin{array}{cc} d & \left(e^{\eta t}\|e\left(x,t,\psi ,\varphi \right){\|}^{\kappa }\right)\\ & =\eta e^{\eta t}{\left\|e\left(x,t,\psi ,\varphi \right)\right\|}^{\kappa }dt+e^{\eta t}\left\{\kappa \right\|e_{1}\left(x,t,\psi ,\varphi \right){\|}^{\kappa -2}\langle e_{1}\left(x,t,\psi ,\varphi \right),d_{1}△e_{1}\left(x,t,\psi ,\varphi \right)-\\ & a_{e}e_{1}\left(x,t,\psi ,\varphi \right)-\left(a_{0}-a_{e}\right)e^{-\vartheta_{1}t}e_{1}\left(x,t,\psi ,\varphi \right)-\beta \left(u_{1}\left(x,t,\psi \right)u_{3}\left(x,t,\psi \right)-u_{1}\left(x,t,\varphi \right)u_{3}\left(x,t,\varphi \right)\right)\rangle dt+\\ & \kappa \|e_{2}\left(x,t,\psi ,\varphi \right){\|}^{\kappa -2}\langle e_{2}\left(x,t,\psi ,\varphi \right),d_{2}△e_{2}\left(x,t,\psi ,\varphi \right)+\beta \left(u_{1}\left(x,t,\psi \right)u_{3}\left(x,t,\psi \right)-u_{1}\left(x,t,\varphi \right)u_{3}\left(x,t,\varphi \right)\right)\\ & -b_{e}e_{2}\left(x.t,\psi ,\varphi \right)-\left(b_{0}-b_{e}\right)e^{-\vartheta_{2}t}e_{2}\left(x,t,\psi ,\varphi \right)\rangle dt+\kappa \|e_{3}\left(x,t,\psi ,\varphi \right){\|}^{\kappa -2}\langle e_{3}\left(x,t,\psi ,\varphi \right),d_{3}△e_{3}\left(x,t,\psi ,\varphi \right)\\ & +ke_{2}\left(x,t,\psi ,\varphi \right)-m_{e}e_{3}\left(x,t,\psi ,\varphi \right)-\left(m_{0}-m_{e}\right)e^{-\vartheta_{3}t}e_{3}\left(x,t,\psi ,\varphi \right)\rangle dt+\frac{1}{2}\kappa \left(\kappa -1\right)\xi_{1}^{2}\left(t\right){\left\|e_{1}\left(x,t,\psi ,\varphi \right)\right\|}^{\kappa }dt\\ & +\frac{1}{2}\kappa \left(\kappa -1\right)\xi_{2}^{2}\left(t\right)\|e_{2}{\left(x,t,\psi ,\varphi \right)\|}^{\kappa }dt+\frac{1}{2}\kappa \left(\kappa -1\right)\xi_{3}^{2}\left(t\right)\|e_{3}{\left(x,t,\psi ,\varphi \right)\|}^{\kappa }dt+\kappa \|e_{1}\left(x,t,\psi ,\varphi \right){\|}^{\kappa -2}\langle e_{1}\left(x,t,\psi ,\varphi \right),\\ & -\xi_{1}\left(t\right)△e_{1}\left(x,t,\psi ,\varphi \right)dB_{1}\left(t\right)\rangle +\kappa \|e_{2}{\left(x,t,\psi ,\varphi \right)\|}^{\kappa -2}\left\langle e_{2}\left(x,t,\psi ,\varphi \right),-\xi_{2}\left(t\right)△e_{2}\left(x,t,\psi ,\varphi \right)dB_{2}\left(t\right)\right\rangle +\\ & \kappa \|e_{1}\left(x,t,\psi ,\varphi \right){\|}^{\kappa -2}\left\langle e_{1}\left(x,t,\psi ,\varphi \right),-\xi_{3}\left(t\right)△e_{3}\left(x,t,\psi ,\varphi \right)dB_{3}\left(t\right)\right\rangle \}\\ & =\eta e^{\eta t}{\left\|e\left(x,t,\psi ,\varphi \right)\right\|}^{\kappa }dt+e^{\eta t}\{-\kappa d_{1}\|e_{1}{\left(x,t,\psi ,\varphi \right)\|}^{\kappa -2}\|\nabla e_{1}{\left(x,t,\psi ,\varphi \right)\|}^{2}-a_{e}{\left\|e_{1}\left(x,t,\psi ,\varphi \right)\right\|}^{\kappa }\\ & -\left(a_{0}-a_{e}\right)e^{-\vartheta_{1}t}\|e_{1}\left(x,t,\psi ,\varphi \right){\|}^{\kappa }-\kappa \beta \|e_{1}\left(x,t,\psi ,\varphi \right){\|}^{\kappa -2}\langle e_{1}\left(x,t,\psi ,\varphi \right),-(u_{1}\left(x,t,\psi \right)e_{3}\left(x,t,\psi ,\varphi \right)+\\ & u_{3}\left(x,t,\varphi \right)e_{1}\left(x,t,\psi ,\varphi \right))\rangle -\kappa d_{2}\|e_{2}{\left(x,t,\psi ,\varphi \right)\|}^{\kappa -2}\cdot \|\nabla e_{2}{\left(x,t,\psi ,\varphi \right)\|}^{2}+\kappa \beta {\left\|e_{1}\left(x,t,\psi ,\varphi \right)\right\|}^{\kappa -2}\\ & \left\langle e_{1}\left(x,t,\psi ,\varphi \right),-\left(u_{1}\left(x,t,\psi \right)e_{3}\left(x,t,\psi ,\varphi \right)+u_{3}\left(x,t,\varphi \right)e_{1}\left(x,t,\psi ,\varphi \right)\right)\right\rangle -b_{e}{\left\|e_{2}\left(x,t,\psi ,\varphi \right)\right\|}^{\kappa }\\ & -\left(b_{0}-b_{e}\right)e^{-\vartheta_{2}t}\|e_{2}\left(x,t,\psi ,\varphi \right){\|}^{\kappa }-\kappa d_{3}\|e_{3}\left(x,t,\psi ,\varphi \right){\|}^{\kappa -2}\|\nabla e_{3}\left(x,t,\psi ,\varphi \right){\|}^{2}+\kappa k{\left\|e_{3}\left(x,t,\psi ,\varphi \right)\right\|}^{\kappa -1}\cdot \\ & \|e_{2}\left(x,t,\psi ,\varphi \right)\|-m_{e}\|e_{3}{\left(x,t,\psi ,\varphi \right)\|}^{\kappa }-\left(m_{0}-m_{e}\right)e^{-\vartheta_{3}t}\|e_{3}\left(x,t,\psi ,\varphi \right){\|}^{\kappa }+\frac{1}{2}\kappa \left(\kappa -1\right)\xi_{1}^{2}\left(t\right){\left\|e_{1}\left(x,t,\psi ,\varphi \right)\right\|}^{\kappa }\\ & +\frac{1}{2}\kappa \left(\kappa -1\right)\xi_{2}^{2}\left(t\right)\|e_{2}{\left(x,t,\psi ,\varphi \right)\|}^{\kappa }+\frac{1}{2}\kappa \left(\kappa -1\right)\xi_{3}^{2}\left(t\right)\|e_{3}{\left(x,t,\psi ,\varphi \right)\|}^{\kappa }\}dt-\kappa e^{\eta t}\xi_{1}\left(t\right){\left\|e_{1}\left(x,t,\psi ,\varphi \right)\right\|}^{\kappa }dB_{1}\left(t\right)\\ & -\kappa e^{\eta t}\xi_{2}\left(t\right)\|e_{2}{\left(x,t,\psi ,\varphi \right)\|}^{\kappa }dB_{2}\left(t\right)-\kappa e^{\eta t}\xi_{3}\left(t\right){\left\|e_{3}\left(x,t,\psi ,\varphi \right)\right\|}^{\kappa }dB_{3}\left(t\right)\\ & \leq \eta e^{\eta t}{\left\|e\left(x,t,\psi ,\varphi \right)\right\|}^{\kappa }dt+\kappa e^{\eta t}\{\beta M_{1}\|e_{1}{\left(x,t,\psi ,\varphi \right)\|}^{\kappa -1}\|e_{3}\left(x,t\psi ,\varphi \right)\|+\beta M_{1}{\left\|e_{1}\left(x,t,\psi ,\varphi \right)\right\|}^{\kappa }+k\\ & \|e_{3}{\left(x,t,\psi ,\varphi \right)\|}^{\kappa -1}\|e_{2}\left(x,t,\psi ,\varphi \right)\|+\frac{1}{2}\kappa \left(\kappa -1\right)\xi_{1}^{2}\left(t\right)\|e_{1}{\left(x,t,\psi ,\varphi \right)\|}^{\kappa }+\frac{1}{2}\kappa \left(\kappa -1\right)\xi_{2}^{2}\left(t\right){\left\|e_{2}\left(x,t,\psi ,\varphi \right)\right\|}^{\kappa }\\ & +\frac{1}{2}\kappa \left(\kappa -1\right)\xi_{3}^{2}\left(t\right)\|e_{3}\left(x,t,\psi ,\varphi \right){\|}^{\kappa }\}dt, \end{array} \end{array}$$

integrate on both sides of the above inequality and take expectations, at the same time, apply the Young inequality, we get

$$\begin{array}{c} \begin{array}{cc} & E[e^{\eta t}\|e\left(x,t,\psi ,\varphi \right){\|}^{\kappa }]\\ & \leq {\left\|e\left(x,0,\psi ,\varphi \right)\right\|}^{\kappa }+E\int_{0}^{t}e^{\eta s}{\left\{\eta \|e\left(x,s,\psi ,\varphi \right)\right\|}^{\kappa }+\left(\kappa -1\right)\|e_{1}{\left(x,s,\psi ,\varphi \right)\|}^{\kappa }+\beta^{\kappa }M_{1}^{\kappa }{\left\|e_{3}\left(x,s,\psi ,\varphi \right)\right\|}^{\kappa }\\ & +\kappa \beta M_{1}\|e_{1}{\left(x,s,\psi ,\varphi \right)\|}^{\kappa }+\left(\kappa -1\right)\|e_{3}{\left(x,s,\psi ,\varphi \right)\|}^{\kappa }+k^{\kappa }{\left\|e_{2}\left(x,s,\psi ,\varphi \right)\right\|}^{\kappa }+\frac{1}{2}\kappa \left(\kappa -1\right)\xi_{1}^{2}\left(t\right)\\ & \|e_{1}{\left(x,t,\psi ,\varphi \right)\|}^{\kappa }+\frac{1}{2}\kappa \left(\kappa -1\right)\xi_{2}^{2}\left(t\right)\|e_{2}{\left(x,t,\psi ,\varphi \right)\|}^{\kappa }+\frac{1}{2}\kappa \left(\kappa -1\right)\xi_{3}^{2}\left(t\right)\|e_{3}\left(x,t,\psi ,\varphi \right){\|}^{\kappa }\}ds. \end{array} \end{array}$$

Next, we take the supremum on both sides of the above inequality

$$\begin{array}{c} E\;\underset{0\leq t\leq T}{\text{sup}}[e^{\eta t}{\left\|e\left(x,t,\psi ,\varphi \right)\right\|}^{\kappa }{]\leq \|e\left(x,0,\psi ,\varphi \right)\|}^{\kappa }+E\underset{0\leq t\leq T}{\text{sup}}M_{5}\int_{0}^{t}e^{\eta s}{\left\|e\left(x,s,\psi ,\varphi \right)\right\|}^{\kappa }ds, \end{array}$$ (17)

here

$M_{5}=max\left\{\eta ,\left(\kappa -1\right)+\kappa \beta M_{1}+\frac{1}{2}\kappa \left(\kappa -1\right)\xi_{1}^{2},k^{\kappa }+\frac{1}{2}\kappa \left(\kappa -1\right)\xi_{2},\left(\kappa -1\right)+\beta^{\kappa }M_{1}^{\kappa }+\frac{1}{2}\kappa \left(\kappa -1\right)\xi_{3}^{2}\right\}$ .

Based on the Gronwall inequality, we obtain

$$\begin{array}{c} {\left\|e\left(x,t,\psi ,\varphi \right)\right\|}^{\kappa }\leq {\left\|e\left(x,0,\psi ,\varphi \right)\right\|}^{\kappa }e^{-\eta t}, \end{array}$$

thereby

$$\begin{array}{c} \underset{t\to \infty }{\text{lim}}\;E{\left\|e\left(x,t,\psi ,\varphi \right)\right\|}^{\kappa }=0. \end{array}$$

Therefore, condition (1) in Lemma 3.6 holds, there exists a stationary distribution for system (8). Next, we prove the uniqueness of stationary distribution, assume that $\bar{\lambda }$ is also a stationary distribution to W(x,t), there exists some constant *M* > 0, We can get the following result

$$\begin{array}{c} |\lambda \left(f\right)-\bar{\lambda }\left(f\right)|\leq \int_{\Omega \times \Omega }\left|P_{t}f\left(\psi \right)-P_{t}f\left(\varphi \right)\right|\lambda \left(d\psi \right)\bar{\lambda }\left(d\varphi \right)\leq Me^{-\eta t}, \end{array}$$

when *t* → ∞, we can get the uniqueness of stationary distribution.

**Remark 6** Theorem 3.7 illustrated the existence and uniqueness of stationary distribution of the solution for the diffusion HBV infection model.

## 4. Numerical simulations

We present the numerical simulation in this section to better understand our results. Based on the Milstein method [25], The system (8) discrete form is as follows:

$$\begin{array}{c} \begin{array}{cc} u_{1(i,j+1)} & =u_{1(i,j)}+[d_{1}\frac{u_{1(i+1,j)}-2u_{1(i,j)}+u_{1(i-1,j)}}{{\left(△x\right)}^{2}}+\lambda -a_{e}u_{1(i,j)}-\left(a_{0}-a_{e}\right)e^{-\vartheta_{1}t}u_{1(i,j)}\\ & -\beta u_{1(i,j)}u_{3(i,j)}]△t-\frac{\xi_{1}}{\sqrt{2\vartheta_{1}}}\sqrt{1-e^{-2\vartheta_{1}\left(j△t\right)}}u_{1(i,j)}\varsigma_{j}-\frac{\xi_{1}^{2}}{4\vartheta_{1}}\left(1-e^{-2\vartheta_{1}\left(j△t\right)}\right)u_{1(i,j)}^{2}\left(\varsigma_{j}^{2}-1\right)△t,\\ u_{2(i,j+1)} & =u_{2(i,j)}+[d_{2}\frac{u_{2(i+1,j)}-2u_{2(i,j)}+u_{2(i-1,j)}}{{\left(△x\right)}^{2}}+\beta u_{1(i,j)}u_{3(i,j)}-b_{e}u_{2(i,j)}-\left(b_{0}-b_{e}e^{-\vartheta_{2}t}\right)\cdot \\ & u_{2(i,j)}]△t-\frac{\xi_{2}}{\sqrt{2\vartheta_{2}}}\sqrt{1-e^{-2\vartheta_{2}\left(j△t\right)}}u_{2(i,j)}\varsigma_{j}-\frac{\xi_{2}^{2}}{4\vartheta_{2}}\left(1-e^{-2\vartheta_{2}\left(j△t\right)}\right)u_{2(i,j)}^{2}\left(\varsigma_{j}^{2}-1\right)△t,\\ u_{3(i,j+1)} & =u_{3(i,j)}+\left[d_{3}\frac{u_{3(i+1,j)}-2u_{3(i,j)}+u_{3(i-1,j)}}{{\left(△x\right)}^{2}}+ku_{2(i,j)}-m_{e}u_{3(i,j)}-\left(m_{0}-m_{e}e^{-\vartheta_{3}t}\right)u_{3(i,j)}\right]△t\\ & -\frac{\xi_{3}}{\sqrt{2\vartheta_{3}}}\sqrt{1-e^{-2\vartheta_{3}\left(j△t\right)}}u_{3(i,j)}\varsigma_{j}-\frac{\xi_{3}^{2}}{4\vartheta_{3}}\left(1-e^{-2\vartheta_{3}\left(j△t\right)}\right)u_{3(i,j)}^{2}\left(\varsigma_{j}^{2}-1\right)△t, \end{array} \end{array}$$

where *ς*_*j*_, (*j* = 1, 2, 3) are independent Gaussian random variables *N*(0, 1). We select the △*t* = 0.1, △*x* = 0.5, *a*_0_ = 0.15, *b*_0_ = 2.6 and *m*_0_ = 0.35, other parameter values are chosen in Table 1:

**Table 1:** Parameter values.

Parameter	Value	Parameter	Value	Parameter	Value
λ	10(*cells ml day*^−1^) [26]	*a*_*e*_	0.1(*day*^−1^) [26]	*ξ*_1_	0.01 [Assumed]
*β*	0.00025(*ml virion day*^−1^) [26]	*ϑ*_1_	0.8[Assumed]	*b*_*e*_	2.4(*day*^−1^) [Assumed]
k	50(*virion cells day*^−1^) [26]	*ξ*_2_	0.03[Assumed]	*m*_*e*_	0.4[Assumed]
*ξ*_3_	0.05[Assumed]	*d*_1_	0.01[Assumed]	*d*_2_	0.03[Assumed]
*d*_3_	0.04[Assumed]	*ϑ*_2_	0.6[Assumed]	*ϑ*_3_	1[Assumed]

initial value: $\left(u_{10}\left(x\right),u_{20}\left(x\right),u_{30}\left(x\right)\right)=\left(sin\frac{\pi x}{60},sin\frac{\pi x}{60},sin\frac{\pi x}{60}\right)$.

### 4.1. The influence of reversion rates for the stationary distribution of the solution

**Fig 1:**
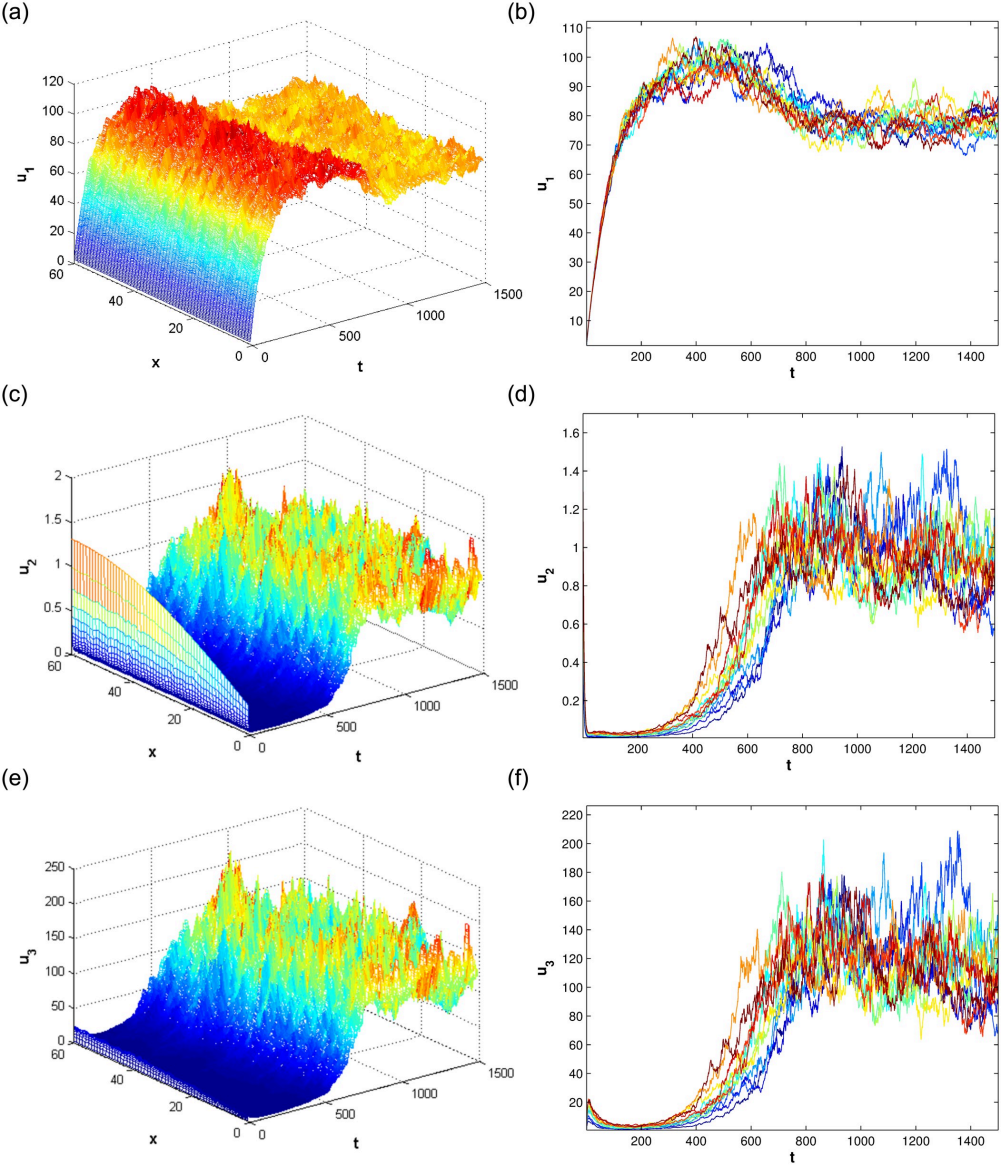
The stationary distribution of the solution for system (8).

**Fig 2:**
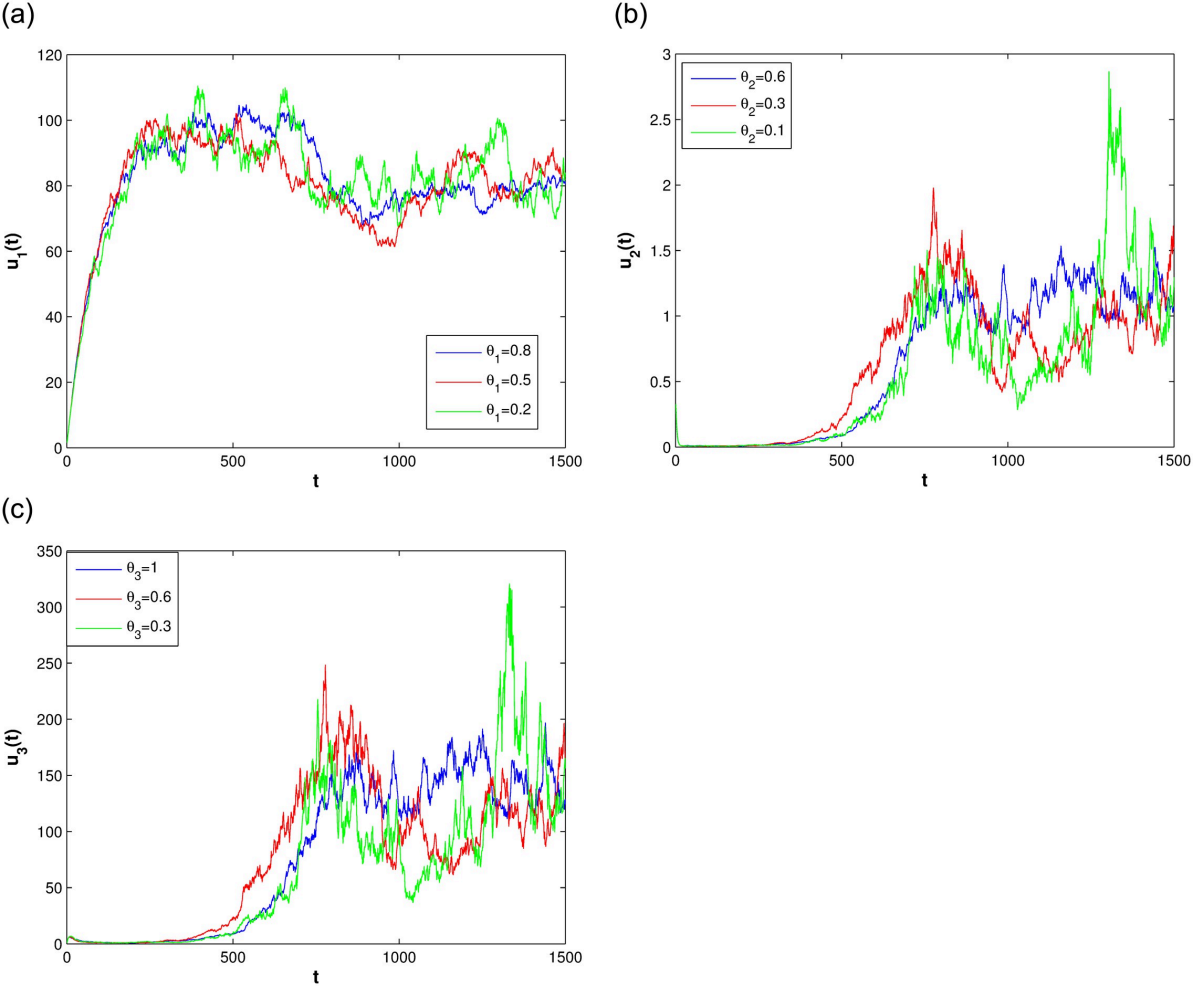
The impact of difference reversion rates for system (8).

**Fig 3:**
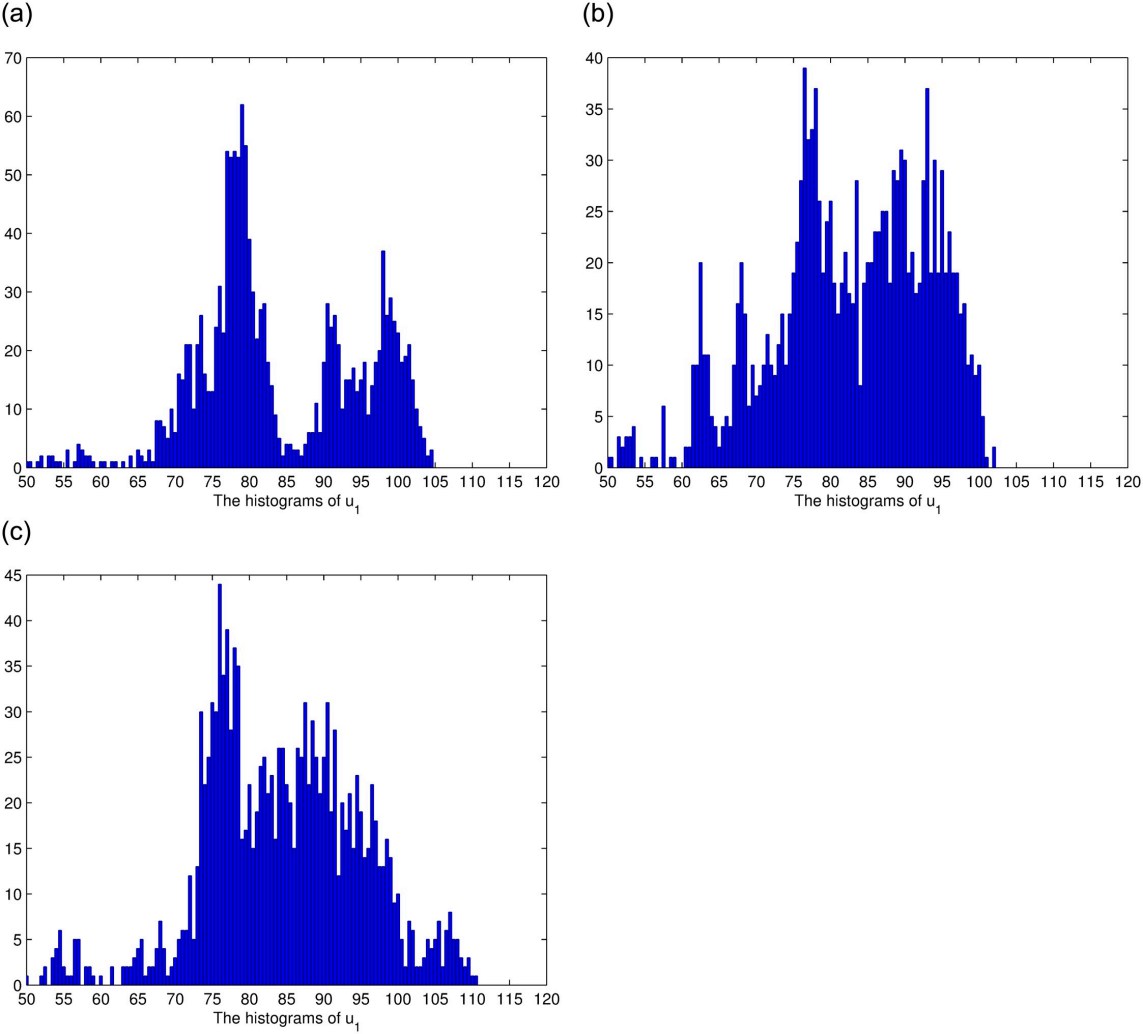
The histograms of *u*_1_ for *θ*_1_ = 0.8, *θ*_1_ = 0.5, *θ*_1_ = 0.2, respectively.

**Fig 4:**
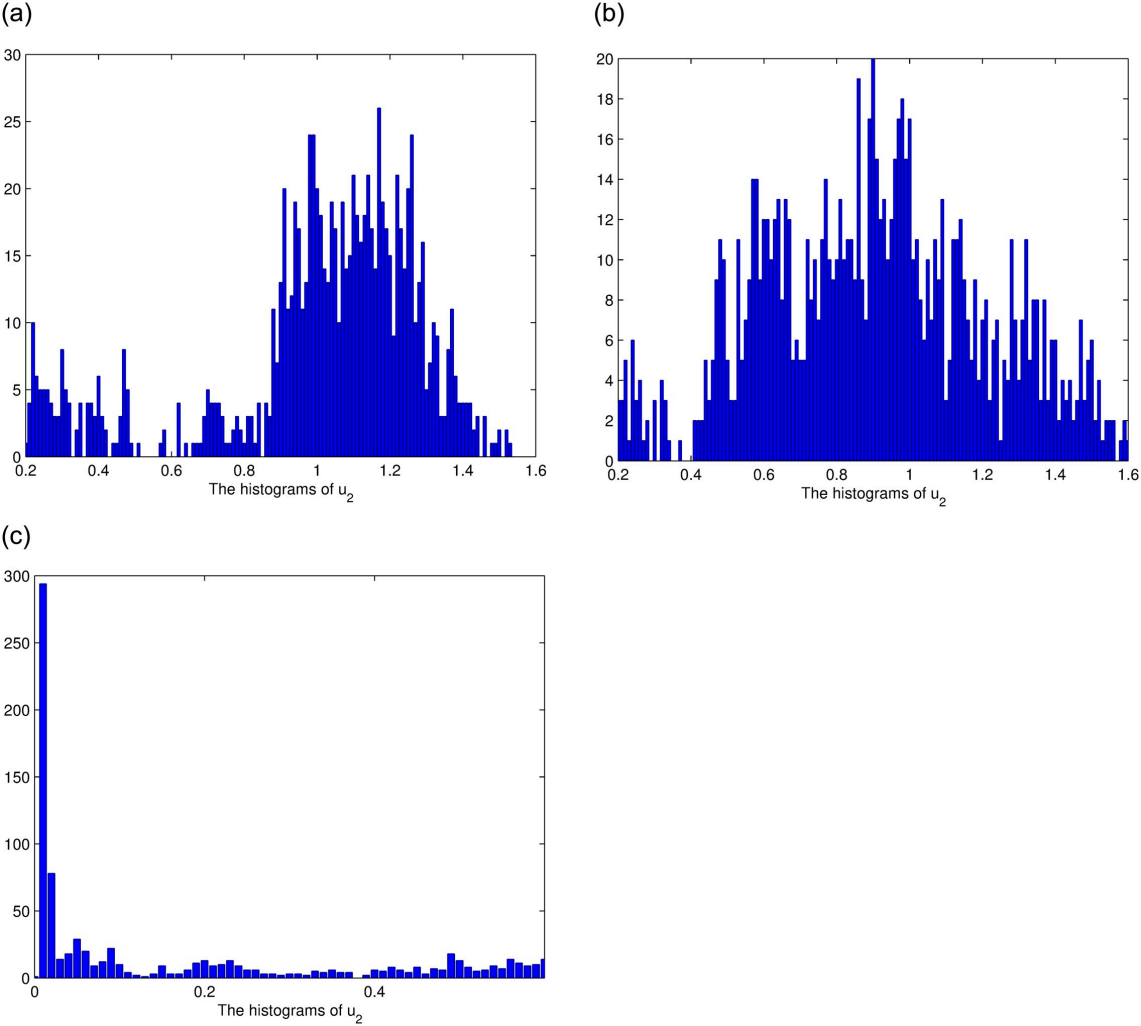
The histograms of *u*_2_ for *θ*_2_ = 0.6, *θ*_2_ = 0.3, *θ*_2_ = 0.1, respectively.

**Fig 5:**
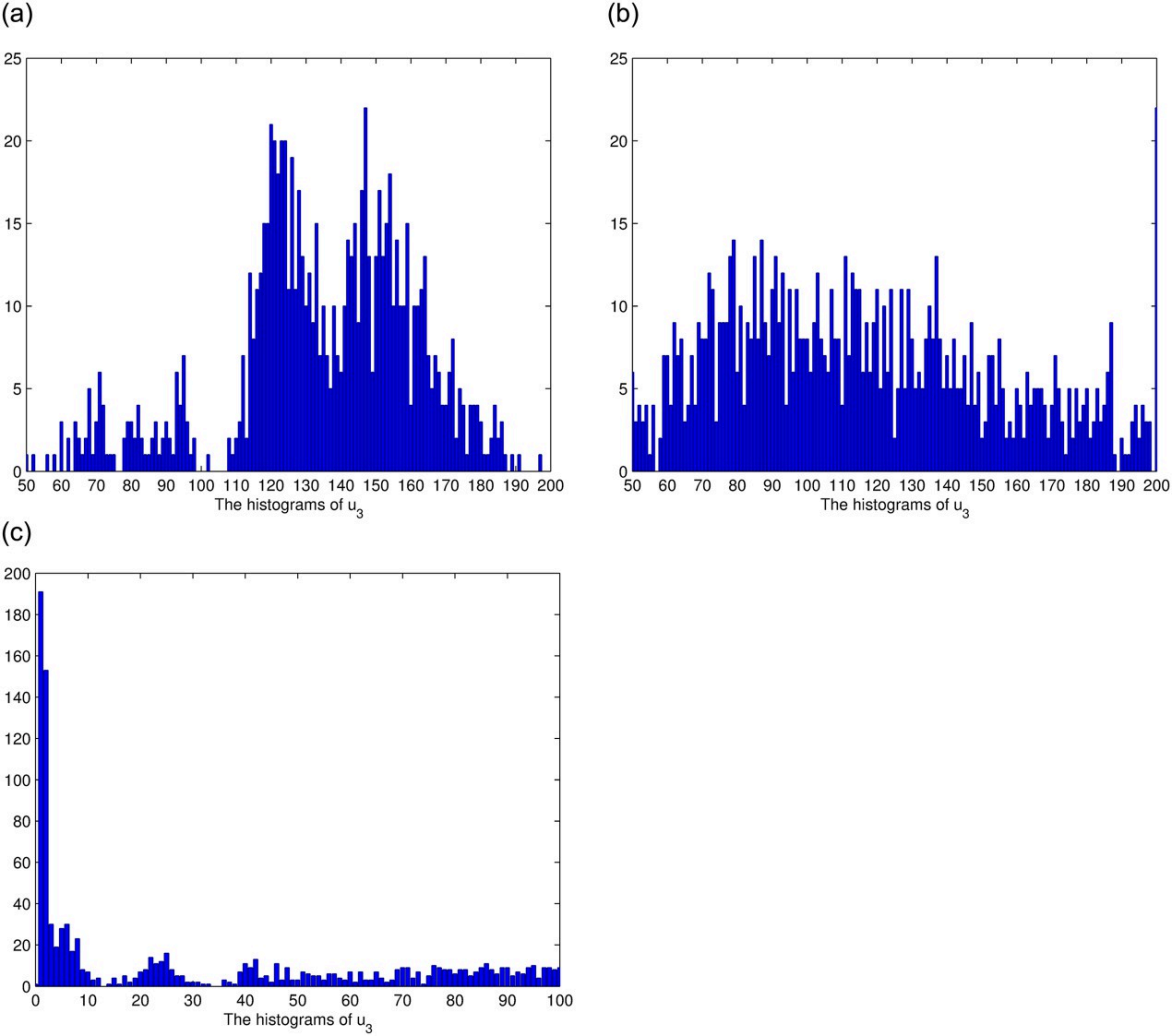
The histograms of *u*_3_ for *θ*_3_ = 1, *θ*_3_ = 0.6, *θ*_3_ = 0.3, respectively.

In this section, we consider the stationary distribution of solution of the system (8). In Fig 1, we can see the existence of the stationary distribution of the solution of system (8). The two-dimensional figure on the right shows the changes in time of the solution in different Spaces, and it can be seen that the stationary distribution of the solution is different in different Spaces. The effect of reversion rates on the solution’s stationary distribution is depicted in Fig 2. For a more intuitive observation of the effect of the response rate in Fig 2, we present Figs 3–5, as the reversion rates, the amplitude of fluctuation becomes smaller, corresponding to the solution distribution being closer to the normal distribution. On the contrary, the smaller the reversion rates, the stronger the vibration and the more dispersed solutions distribution.

### 4.2. Impact of noise intensity for stationary distribution of solution

**Fig 6:**
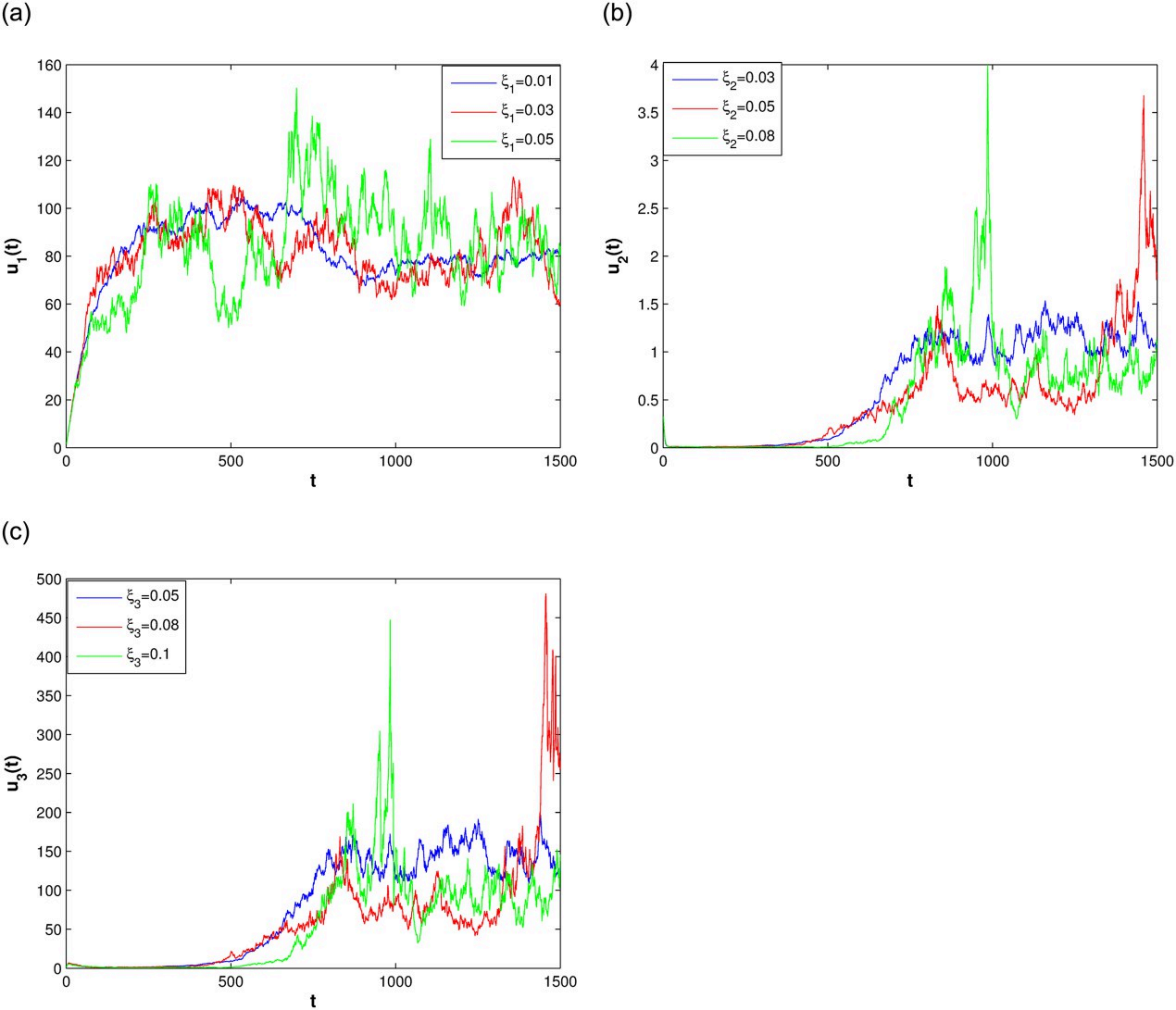
The impact of difference noise intensity for system (8).

**Fig 7:**
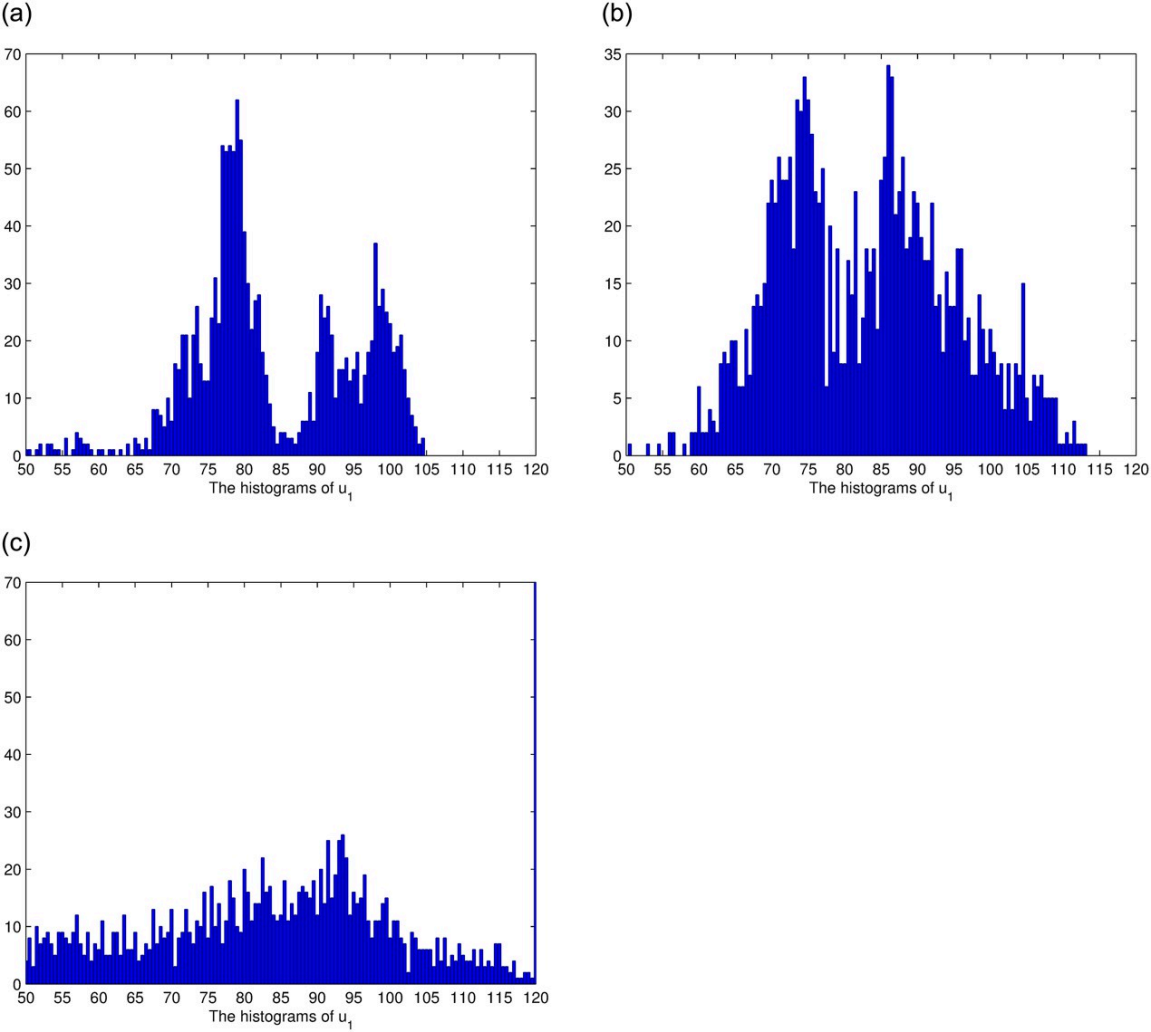
The histograms of *u*_1_ for *ξ*_3_ = 0.01, *ξ*_3_ = 0.03, *ξ*_3_ = 0.05, respectively.

**Fig 8:**
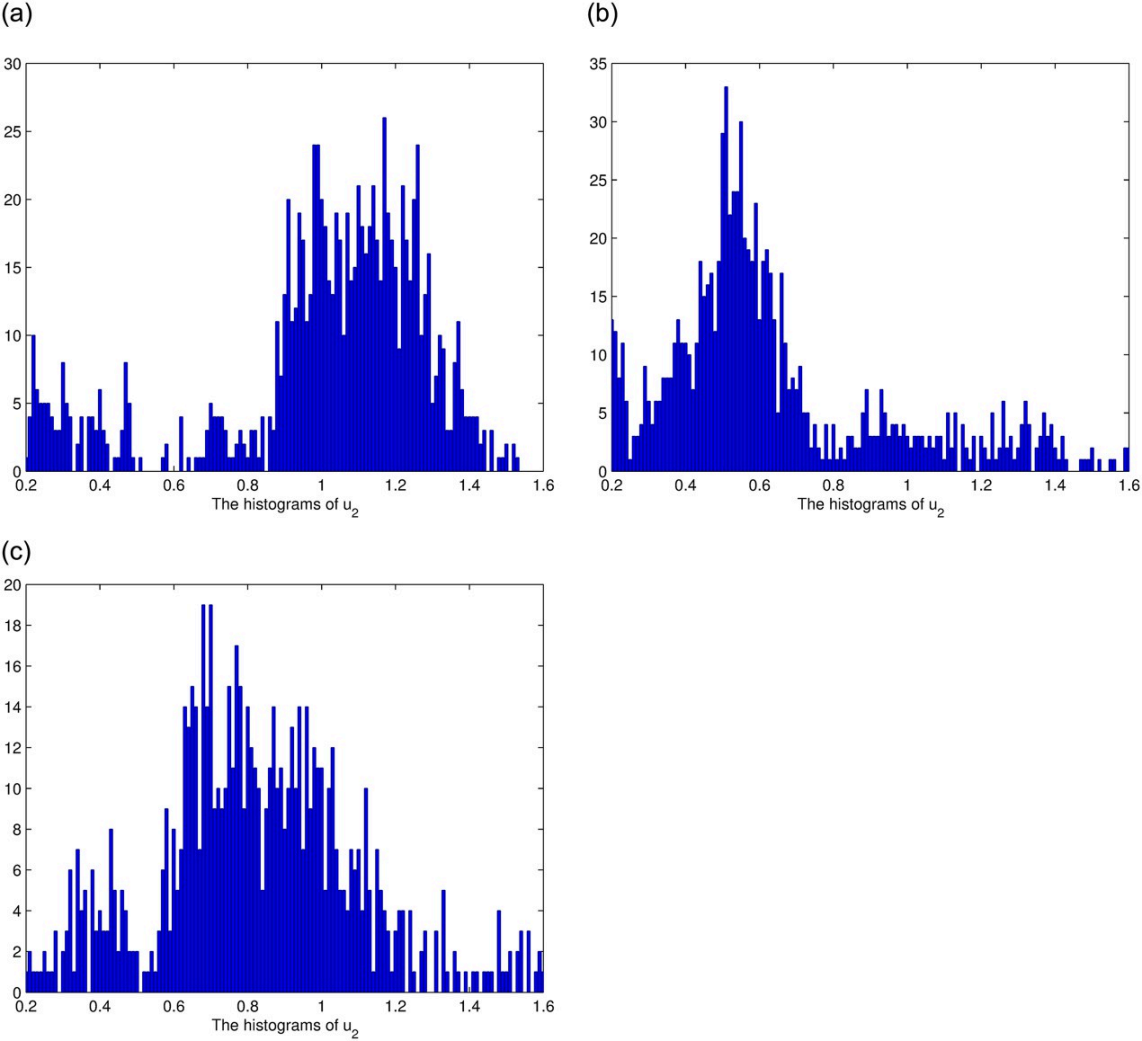
The histograms of *u*_2_ for *ξ*_3_ = 0.03, *ξ*_3_ = 0.05, *ξ*_3_ = 0.08, respectively.

**Fig 9:**
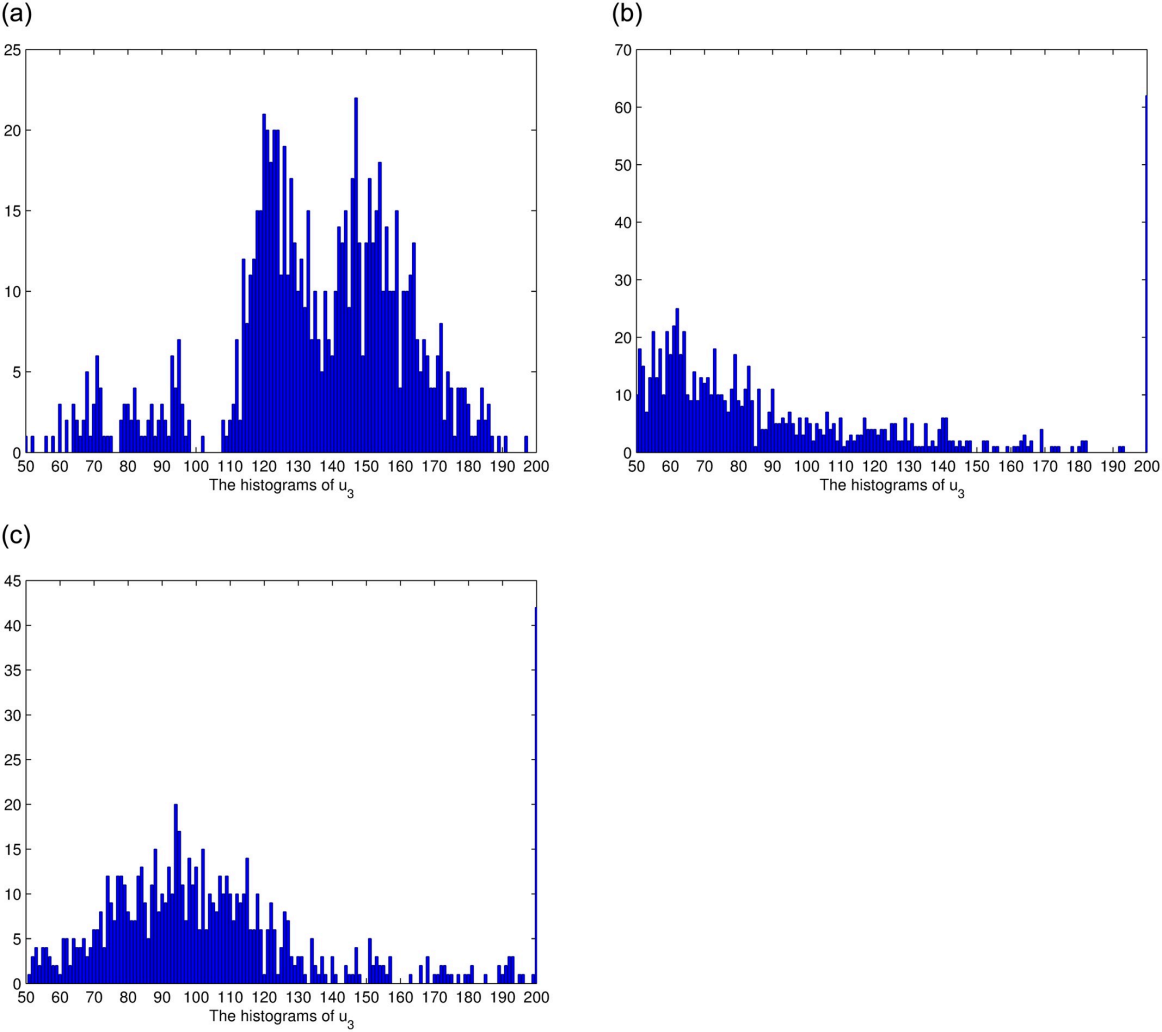
The histograms of *u*_3_ for *ξ*_3_ = 0.05, *ξ*_3_ = 0.08, *ξ*_3_ = 0.1, respectively.

This section considers the influence of noise intensity on the stationary distribution of solutions. The image fluctuation decreases as the noise intensity decreases (Fig 6), for ease of observation, we present the histograms of *u*_1_, *u*_2_, *u*_3_ for each case in Fig 6, and it can be seen that the smaller the noise, the closer the solution is to the normal distribution [see Figs 7–9].

## 5. Conclusions

Mathematical models are regarded as an efficient method when it comes to comprehending how HBV is transmitted. In recent years, many papers have investigated the dynamical behavior of the model, among which we list Din and Li [6], Ge et al. [11], Wu and Zou [16] and other related literatures. However, the models in these literature are all derived from ordinary differential equations, or only one that considers the diffusion of cells and viruses, ignoring the simultaneous migration of cells and viruses, that is, spatial diffusion.

This study investigated a stochastic HBV infection model combined with diffusion of cells and viruses and the mean-reverting Ornstein-Uhlenbeck process. We first demonstrate the stationary distribution of the solution to the diffusion model of the HBV infection was shown to exist and be unique under sufficient conditions. The influence of reversion rates and noise intensity on the disease is shown, the higher the reversion rates and the smaller the noise, the closer the solution is to the normal distribution. Therefore, increasing the reversion rates and reducing the influence of random factors are beneficial to the treatment of the disease. Meanwhile, the stationary distribution means the disease will persist long-term once infected. Because the system may be disrupted by impulsive perturbations, Markov switching, Lévy jumps, and other random factors, it remains a problem that requires further investigation. We will explore these issues in our future work.
