# Supplementary material for: Stationary distribution of a reaction-diffusion hepatitis B virus infection model driven by the Ornstein-Uhlenbeck process
Source: PLoS One. 2023 Sep 29;18(9):e0292073. doi: 10.1371/journal.pone.0292073 (PMC10540978; doi:10.1371/journal.pone.0292073)
Supplement: Attachment — Submitted filename: Report _5.pdf [file pone.0292073.s001.pdf]

Report on ”Stationary distribution of a reaction-diffusion hepatitis B virus infection model driven by the Ornstein-Uhlenbeck process”

This paper studies a reaction-diffusion hepatitis B virus (HBV) infection model based on the mean-reverting Ornstein-Uhlenbeck process. It can be accepted for the publication after some major revisions.

Comments

1. In the abstract, use the present time. For example, change ”We demonstrated” to ”We demonstrate”.
2. Add a reference for the statistical data given in the first paragraph of the introduction section.
3. Study the stability analysis of your model.
4. Check the proofs of Theorems.
5. Give the biological meanings of the results given in each Theorem.
6. Compare your model and results with others existing in the literature.
7. Check the language and punctuation of the manuscript.
8. Check and unify the citation of references.
