# Supplementary material for: Stationary distribution of a reaction-diffusion hepatitis B virus infection model driven by the Ornstein-Uhlenbeck process
Source: PLoS One. 2023 Sep 29;18(9):e0292073. doi: 10.1371/journal.pone.0292073 (PMC10540978; doi:10.1371/journal.pone.0292073)
Supplement: Attachment — Submitted filename: Report.pdf [file pone.0292073.s002.pdf]

Review report

RE : Stationary distribution of a reaction-diffusion hepatitis B virus infection model driven by the Ornstein-Uhlenbeck process

The authors proposed a reaction-diffusion hepatitis B virus (HBV) infection model based on the mean-reverting Ornstein-Uhlenbeck process. They investigated the qualitative and quantitative behavior of the model.

Comments:

(1) In the introduction the authors need to clarify more why the mean-reverting Ornstein-Uhlenbeck process has better characteristics than white noise in biological models.

(2) In Page 2; the statement " It was concluded that the solution tends more toward the stationary distribution, the higher the fluctuation and the lower the noise." should be clarified more.

(3) What is the biological interpretation for the stationary distribution property?

(4) The numerical simulations part should be revised in terms of the following points: a) the discussion for subsections 4.1 & 4.2 are not clear and should be improved, b) the captions of all figures should be revised.

(5) In Conclusion section, the authors should interpret the main results, place them in context of previous findings.

(6) In the introduction, it is recommended to mention the names of authors or simply "In [11,12]...." rather than i.e. Before model (1) "References [11,12]...." ; In addition, the primary goals of the paper can be organized in a better way.

(7) Check the statement after (4) "the mean-revertingmeanreverting".

(8) The paper needs proofreading.
