# Supplementary material for: Stationary distribution of a reaction-diffusion hepatitis B virus infection model driven by the Ornstein-Uhlenbeck process
Source: PLoS One. 2023 Sep 29;18(9):e0292073. doi: 10.1371/journal.pone.0292073 (PMC10540978; doi:10.1371/journal.pone.0292073)
Supplement: Attachment — Submitted filename: Response-to-reviewers.pdf [file pone.0292073.s003.pdf]

Response to reviewers on "Stationary distribution of a reaction-diffusion hepatitis B virus infection model driven by the Ornstein-Uhlenbeck process"

Authors: Zhenyu Zhang, Guizhen Liang, Kangkang Chang.

Manuscript Number: PONE-D-23-1283

Dear editor:

Thank you for the opportunity to revise my article entitled "Stationary distribution of a reaction-diffusion hepatitis B virus infection model driven by Ornstein-Uhlenbeck process"(Manuscript ID: PONE-D-23-1283). Through personal negligence, we made some mistakes and wrote some unclear sentences in the original manuscript. The editor and reviewers have pointed out many mistakes and corrected them, for which we express our great appreciation. The reviewers made some good suggestions which I address below.

Part A Reviewer 1:

(1) In the introduction the authors need to clarify more why the mean-reverting Ornstein-Uhlenbeck process has better characteristics than white noise in biological models.

Response: Thank you very much for your advice, we have made some modifications, please see the line 23, line 24, line 25 and line 26 on page 2 in the revised manuscript.

(2) In Page 2; the statement It was concluded that the solution tends more toward the stationary distribution, the higher the fluctuation and the lower the noise. should be clarified more.

Response: Thank you very much for your advice, we want to express is"the higher the reversion rate and the lower the noise". Meanwhile, we have made some modifications, please see the line 32 and line 33 on page 2 in the revised manuscript.

(3) What is the biological interpretation for the stationary distribution property?

Response: Thank you very much for your advice. The biological interpretation for the stationary distribution is that the disease will persist long-term once infected. Please see the line 13 in the Conclusions.

(4) The numerical simulations part should be revised in terms of the following points: a) the discussion for subsections 4.1 and 4.2 are not clear and should be improved, b) the captions of all figures should be revised.

Response:Thank you very much for your advice, we have made some modifications, please see the subsections 4.1, subsections 4.2 and the captions of all figures in the revised manuscript.

(5) In Conclusion section, the authors should interpret the main results, place them in context of previous findings.

Response:Thank you very much for your advice, because we did not express it clearly, it caused inconvenience in understanding. We have made some modifications, please see the conclusions.

(6) In the introduction, it is recommended to mention the names of authors or simply In [11,12].... rather than i.e. Before model (1) References [11,12].... ; In addition, the primary goals of the paper can be organized in a better way.

Response:Thank you very much for your advice, we have made some modifications, please see the line 9 on page 2 in the revised manuscript. Meanwhile, we reorganized the primary goals of the paper, please see the line 28, line 29 and line 30 on page 2.

(7) Check the statement after (4) the mean-revertingmeanreverting.

Response:Thank you very much for your advice, we have made some modifications, please see the line 16 on page 3 in the revised manuscript.

(8) The paper needs proofreading.

Response:Thank you very much for your advice, we've checked the proof of the theorem and found that there are mistakes in the writing, such as

In Lemma 3.1, $B_1\dot{}(t)$, $B_2\dot{}(t)$, $B_2\dot{}(t)$ should be $\dot{B}_1(t)$, $\dot{B}_2(t)$, $\dot{B}_2(t)$;

Line 8 and line 9 in Theorem 3.5, $\|u_2(x,s)^2\|$ and $\|u_3(x,s)^2\|$ should be $\|u_2(x,s)\|^2$ and $\|u_3(x,s)\|^2$;

Line 5 page 12, $a_e e_1(x,t,\psi.\varphi)$ should be $a_e e_1(x,t,\psi,\varphi)$;

Line 9 and line 18 page 12, $\|e_2(x,t,\psi,\varphi)^\kappa\|$ and $\|e_2(x,t,\psi,\varphi)^\kappa\|$ should be $\|e_2(x,t,\psi,\varphi)\|^\kappa$ and $\|e_2(x,t,\psi,\varphi)\|^\kappa$;

Punctuation was introduced into each formula.

Part B Reviewer 2:

(1) In the abstract, use the present time. For example, change "We demonstrated" to "We demonstrate".

Response:Thank you very much for your advice, we have made some modifications, please see the line 2 in the abstract.

(2) Add a reference for the statistical data given in the first paragraph of the introduction section.

Response:Thank you very much for your advice, we have added the source of relevant data, please see reference 1 and reference 2.

(3) Study the stability analysis of your model.

Response: Thank you very much for your advice. We can obtain that the model is finite-time stable, please see the Theorem 3.4 in the revised manuscript.

(4) Check the proofs of Theorems.

Response:Thank you very much for your advice, we've checked the proof of the theorem and found that there are mistakes in the writing, such as

In Lemma 3.1, $B_1^{\cdot}(t)$, $B_2^{\cdot}(t)$, $B_2^{\cdot}(t)$ should be $\dot{B}_1(t)$, $\dot{B}_2(t)$, $\dot{B}_2(t)$;

Line 8 and line 9 in Theorem 3.5, $\|u_2(x,s)^2\|$ and $\|u_3(x,s)^2\|$ should be $\|u_2(x,s)\|^2$ and $\|u_3(x,s)\|^2$;

Line 5 page 12, $a_e e_1(x,t,\psi.\varphi)$ should be $a_e e_1(x,t,\psi,\varphi)$;

Line 9 and line 18 page 12, $\|e_2(x,t,\psi,\varphi)^\kappa\|$ and $\|e_2(x,t,\psi,\varphi)^\kappa\|$ should be $\|e_2(x,t,\psi,\varphi)\|^\kappa$ and $\|e_2(x,t,\psi,\varphi)\|^\kappa$.

(5) Give the biological meanings of the results given in each Theorem.

Response: Thank you very much for your advice, we have made some modification, please see the Remark 1, Remark 2, Remark 3, Remark 4, Remark 5, Remark 6 in the revised manuscript.

(6) Compare your model and results with others existing in the literature.

Response:Thank you very much for your advice. In the conclusion, we present a comparison between the model and the results.

(7) Check the language and punctuation of the manuscript.

Response: Thank you very much for your advice, we have checked the language of

the manuscript, for example, line 14 page 2, line 5 page 4, and the punctuation of the manuscript. If anything is not suitable, we will revise it again.

(8) Check and unify the citation of references.

Response: Thank you very much for your advice, we have unified the citation of references, please see the revised manuscript.

We have made appropriate changes in the revised paper, which I submit, along with this response.

We look forward to hearing from you soon.

Yours sincerely,

Guizhen Liang
